# Dissipate and Recentre (D&R) experimental campaign on real scale GFRP composite frame structures: data set

**DOI:** 10.12688/openreseurope.20500.2

**Published:** 2026-01-14

**Authors:** José Gonilha, João Ramôa Correia, Manuela Buttazzi, Francesco Ciani, Francisco Javier Molina, Simone Peloso

**Affiliations:** 1 IDMEC, Instituto Superior Técnico, Universidade de Lisboa, Lisbon, Portugal; 2 DECivil, Instituto Superior Técnico, Universidade de Lisboa, Lisbon, Portugal; 3 CERIS, Instituto Superior Técnico, Universidade de Lisboa, Lisbon, Portugal; 4 MM s.r.l. a Socio Unico, Udine, Italy; 5 European Commission Joint Research Centre Ispra, Ispra, Lombardy, Italy

**Keywords:** GFRP, Seismic design, Eurocode, Cyclic testing, Large-scale tests

## Abstract

In the scope of the Dissipate&Recentre project, which was granted access to the ELSA Reaction Wall of the Joint Research Centre physical research infrastructure of the European Commission, large scale composite frame structures were tested under lateral loading. The test specimens, comprising pultruded glass fibre reinforced polymer (GFRP) profiles, connected with stainless steel elements, had two longitudinal (test direction) bays, two storeys and a shorter transverse bay. Concrete slabs were provided as flooring system, simulating a quasi-permanent load level – as that used in seismic design. A total of 4 full-scale specimens were tested, two including a bracing system, also comprising pultruded GFRP profiles, and two without bracings. For each configuration, one specimen was tested under monotonic loading and the other under cyclic loading. The aim of this paper is to disseminate the experimental data obtained in these tests. To that end, the experimental campaign is thoroughly described, including all the relevant details of the test specimens, the construction process, the setup and instrumentation, and the test programme. Additionally, a brief description of the test observations is also provided. The test results showed that the structural integrity of these GFRP frames was maintained up to 2% inter-storey drifts, for both braced and unbraced configurations, presenting very limited damage. These results indicate that GFRP frame structures may be designed to withstand significant seismic actions.

## Introduction

Over the past decades, fibre reinforced polymer (FRP) composites have been increasingly used as structural materials in the construction industry, owing to their high strength, low self-weight, non-corrodibility and reduced maintenance costs ^1^. Among FRP composites, pultruded Glass-FRP (GFRP) profiles are an especially attractive solution for new construction and rehabilitation, due to their competitive cost. Moreover, their high durability guarantees long service life and bolted connections enable the design of demountable structures, thus promoting the sustainability of the construction industry and aligning with the priorities of the European Commission (Green Deal and New European Bauhaus) and of the Joint Research Centre (JRC) infrastructure (design and retrofit for resilience, new materials and technologies, and sustainable materials for construction).

On the other hand, pultruded GFRP profiles typically present brittle failure modes, which contrast with current design philosophies that aim at exploiting material (and system) ductility. This has raised well-founded concerns about the application of GFRP profiles in seismic areas, which is aggravated by the lack of comprehensive seismic design guidance. Previous research conducted at IST-ULisboa has addressed this concern, through the development of improved beam-to-column bolted connections ^2– 6^, and the study of two-dimensional ^7, 8^ and three-dimensional ^9^ pultruded GFRP frames.

This paper presents the data set of the Dissipate and Recentre (D&R) project, which aims at experimentally investigating the sway behaviour of large-scale GFRP frames. The objectives of the project are to assess the behaviour of these structures under lateral loading, assessing and proposing seismic design methods and guidelines. The experimental campaign was performed at the European Laboratory for Structural Assessment (ELSA) of the JRC, and the User Access Team comprises well-established senior experts, several young researchers and an industrial partner (SME). The members of the proposing team have a leading role in ongoing efforts for the development of European standards for the design of FRP structures ^10^, for which the results of the project can provide essential knowledge. Such standards will support the market uptake of this innovative technology in the construction sector.

The test specimens consisted of 2-storey frames, with 2 longitudinal bays and a single transverse bay. The structures were made of pultruded GFRP profiles, with stainless-steel connections. Concrete precast slabs were installed on the GFRP beams creating the floors of the specimens, simulating the equivalent vertical loads of a real application, and allowing to anchor the actuators. The experimental campaign included the testing of four specimens: two without lateral bracings and two with such bracings. The modal identification of all specimens was performed using data of snap-back tests. For both types of frames, the first specimen was tested under monotonic loading, while the second underwent cyclic pseudo-static loading with increasing displacement amplitude. All tests were performed under displacement control. During the tests, the monitoring system recorded the applied loads and displacements, as well as rotations and strains of joints and members at key locations.

## The experimental campaign

**Figure 1.**
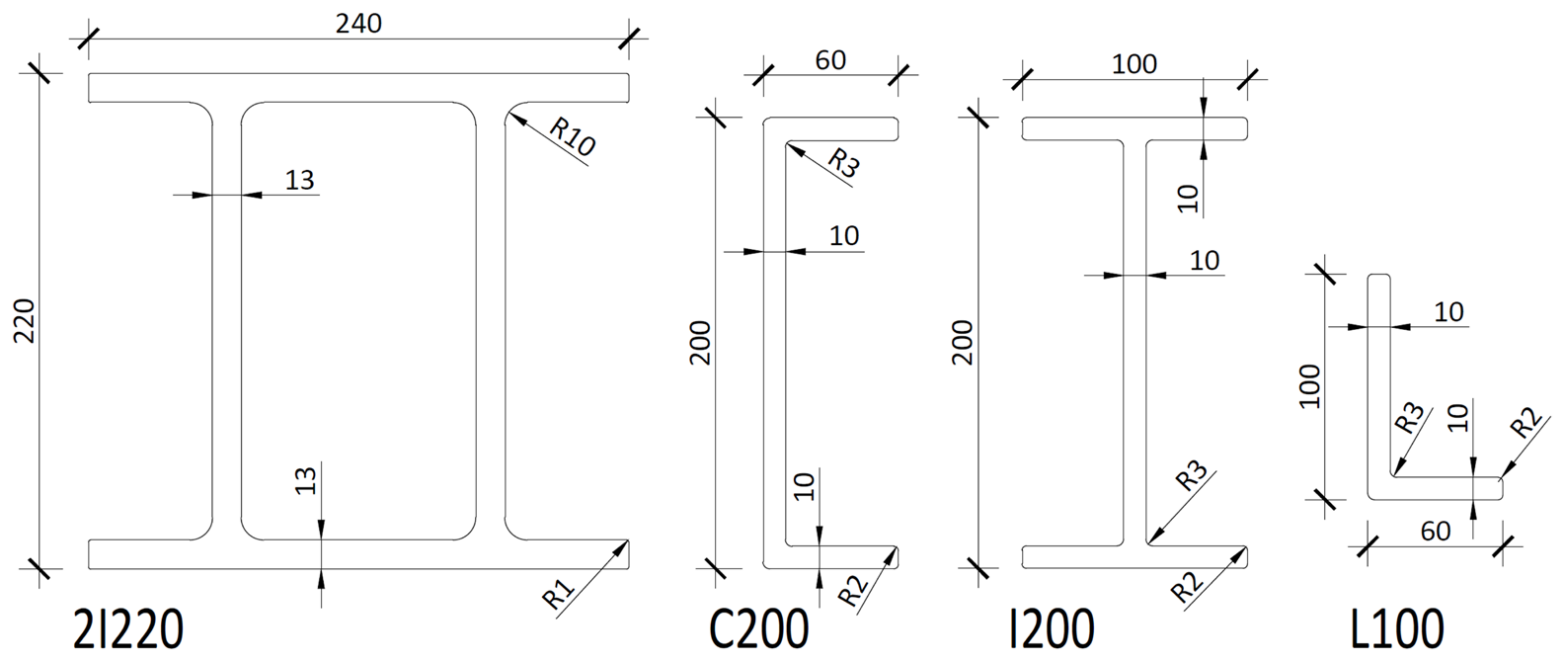
Cross-sections of the pultruded GFRP profiles used in the tests (dimensions in mm).

The experimental campaign consisted of sway tests of large-scale frame structures comprising pultruded GFRP profiles, as shown in Figure 1. The columns consisted of double-I section profiles (220×240×13 mm), and the longitudinal beams were made of two channel section profiles (200×60×10 mm). The transverse beams, on the other hand, consisted of I-shaped profiles (200×100×10 mm). Finally, in the braced frames, the bracings were made of angle (L-shaped) section profiles (100×60×10 mm).

### Materials

The pultruded GFRP profiles used for the specimens were supplied by MM s.r.l. a Socio Unico, which is part of the User Assess Team. All profiles used are classified as “CE-A” quality (internal commercial reference ^11^) and the mechanical properties reported by the manufacturer are presented in Table 1.

**Table 1.** GFRP material properties ^11^.

Loading	Property	Direction	Symbol	Values		Unit
				Average	Characteristic	
In-plane tension	Modulus	Longitudinal	*E _x,t_*	33.1	-	GPa
		Transverse	*E _y,t_*	11.8	-	
	Strength	Longitudinal	*f _x,t_*	470	451	MPa
		Transverse	*f _y,t_*	90.1	84.6	
	Poisson’s ratio	Major	*υ _xy_*	0.30	-	-
		Minor	*υ _yx_*	0.10	-	
In-plane compression	Modulus	Longitudinal	*E _x,c_*	30.0	-	GPa
		Transverse	*E _y,c_*	11.1	-	
	Strength	Longitudinal	*f _x,c_*	393	374	MPa
		Transverse	*f _y,c_*	127	115	
Flexure	Strength	Longitudinal	*f _x,f_*	494	472	MPa
		Transverse	*f _y,f_*	217	185	
Interlaminar shear	Strength	Longitudinal	*f _xz,ILS_*	25.1	21.3	MPa
Pin-bearing	Strength	Longitudinal	*f _x,br_*	225	200	MPa
		Transverse	*f _y,br_*	155	122	

The connections between the columns and the footings (anchored to the laboratory’s strong floor) comprised two gusset plates (8 mm thickness) welded to a base plate (15 mm thickness), all made of 1.4301 ^12^ (AISI 304 ^13^) stainless steel. The web cleated connections between the columns and the transverse beams were also produced by cold forming 6 mm thick 1.4301 ^12^ stainless steel plates. The gusset and base plates connecting the columns to the bracings (braced specimens only) were made of 1.4301 stainless steel ^12^. All connections between GFRP members were bolted using A2-70 ^14^ grade stainless steel bolts, rods, washers and nuts. The column-to-footing connection plates were anchored to the footings with *ϕ*16 mm carbon steel rods, class 10.9 ^15^.

A precast flooring system was installed on the GFRP frame specimens, materialized by C30/37 ^16^ concrete and reinforced with carbon steel rebars class B450C ^17^. It should be stressed that the concrete slabs are not part of the conceptual design of the specimens (in an actual all-composite structure, the floors would likely be made of composite sandwich panels), serving only as a mean to apply vertical (dead) and horizontal (connected to the actuator) loads without suffering any damage during the tests.

### Test specimens

Four specimens were tested in the experimental campaign, as summarized in Table 2. The specimens can be divided in two configuration categories - (i) braced and (ii) unbraced - and in two test categories - (a) monotonic and (b) pseudo-static cyclic loading (henceforth referred to as “cyclic”).

**Table 2.** List of test specimens.

Specimen	Bracings	Loading
uFrame-M	No	Monotonic
uFrame-C	No	Cyclic
BFrame-M	Yes	Monotonic
BFrame-C	Yes	Cyclic

All specimens had the same overall geometry, detailed in the next subsection, with specimens BFrame-M and BFrame-C containing a lateral bracing system.

**Figure 2.**
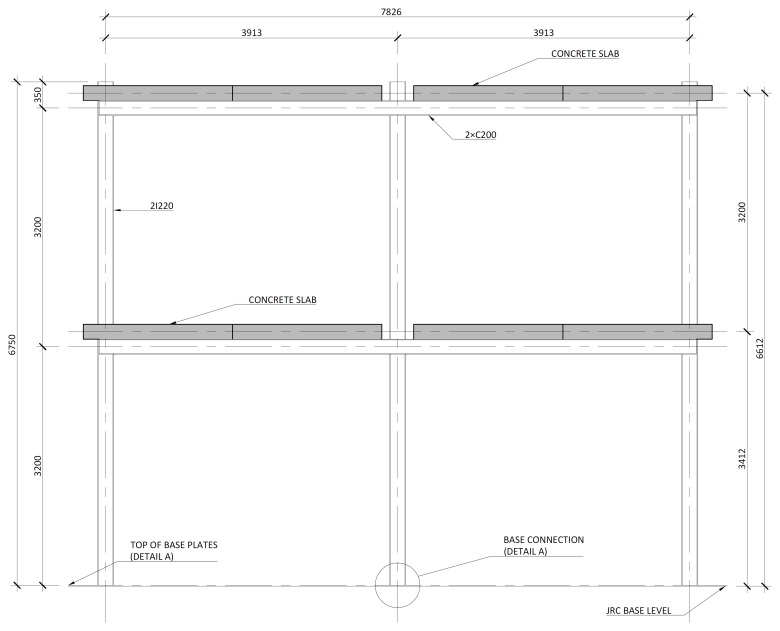
Elevation view of unbraced specimens (dimensions in mm).

***Geometry of the specimens.*** Figure 2 shows the lateral elevation view of an unbraced specimen. The GFRP frames comprised two longitudinal bays, with 3913 mm between column axes. The total height of the columns was 6750 mm, with the axis of the 1 ^st^ storey beams being positioned at 3200 mm from the bottom of the columns, and the 2 ^nd^ storey beams positioned at 6400 mm.

**Figure 3.**
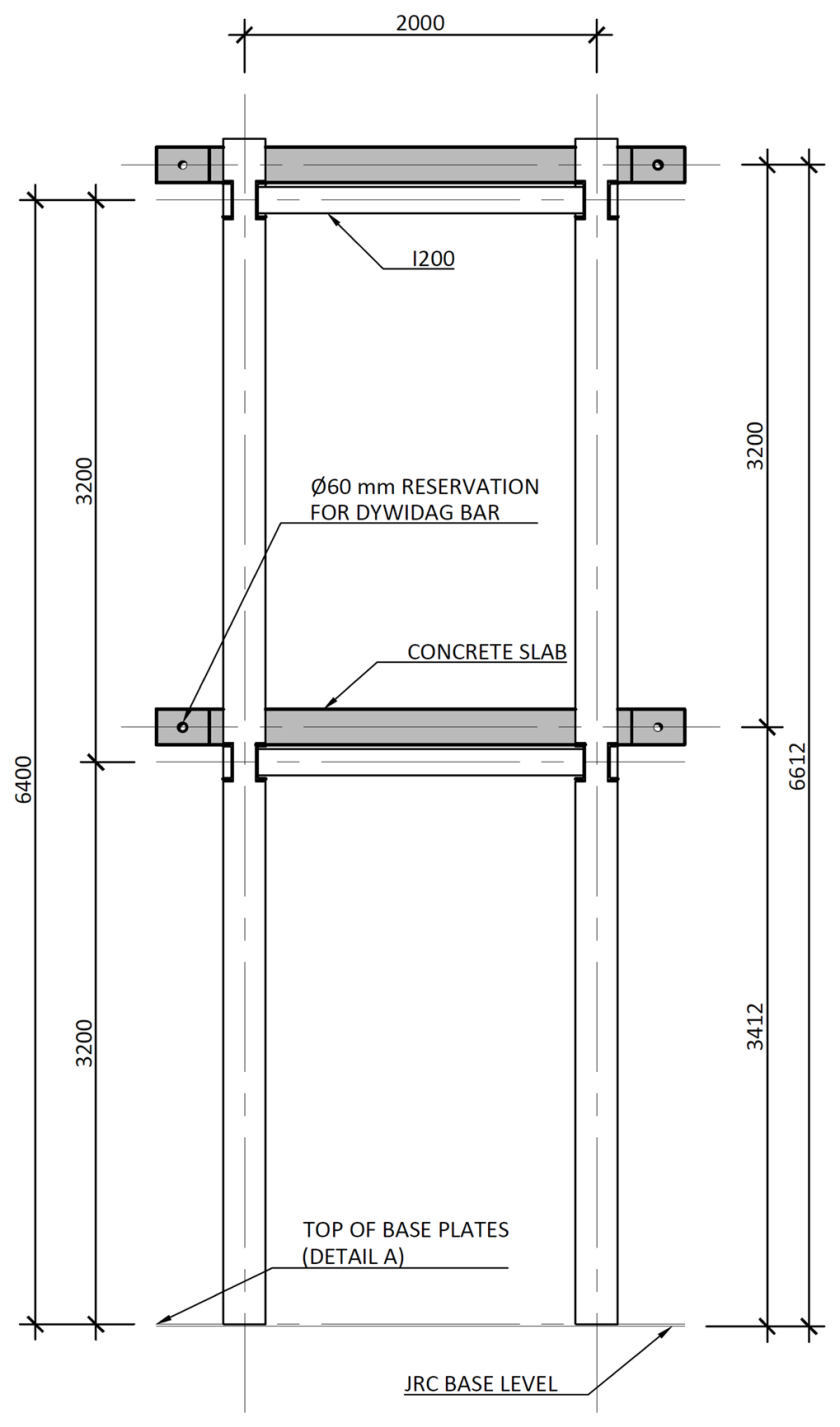
Side view of specimens (dimensions in mm).

Figure 3 illustrates the side view of the specimens, showing the single transverse bay with 2000 mm between the column axes.

**Figure 4.**
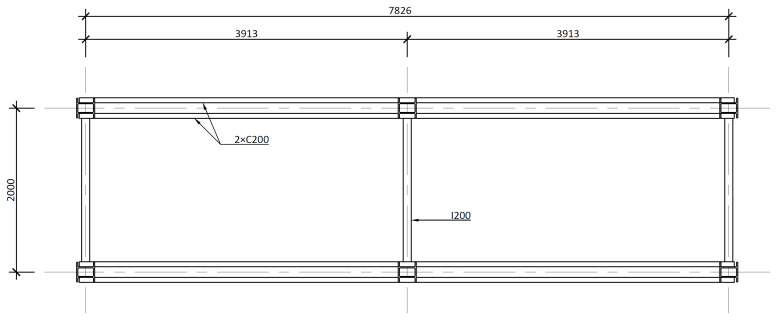
Plan view of specimens without floor slabs (dimensions in mm).

**Figure 5.**
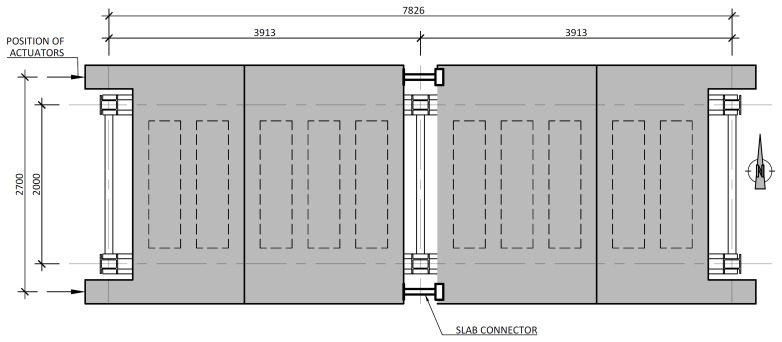
Plan view of specimens with floor slabs (dimensions in mm).

Figure 4 and Figure 5 show the plan view of specimens with and without the slabs installed. The latter figure includes the position of the slab connectors, whose geometry is detailed ahead in this document.

**Figure 6.**
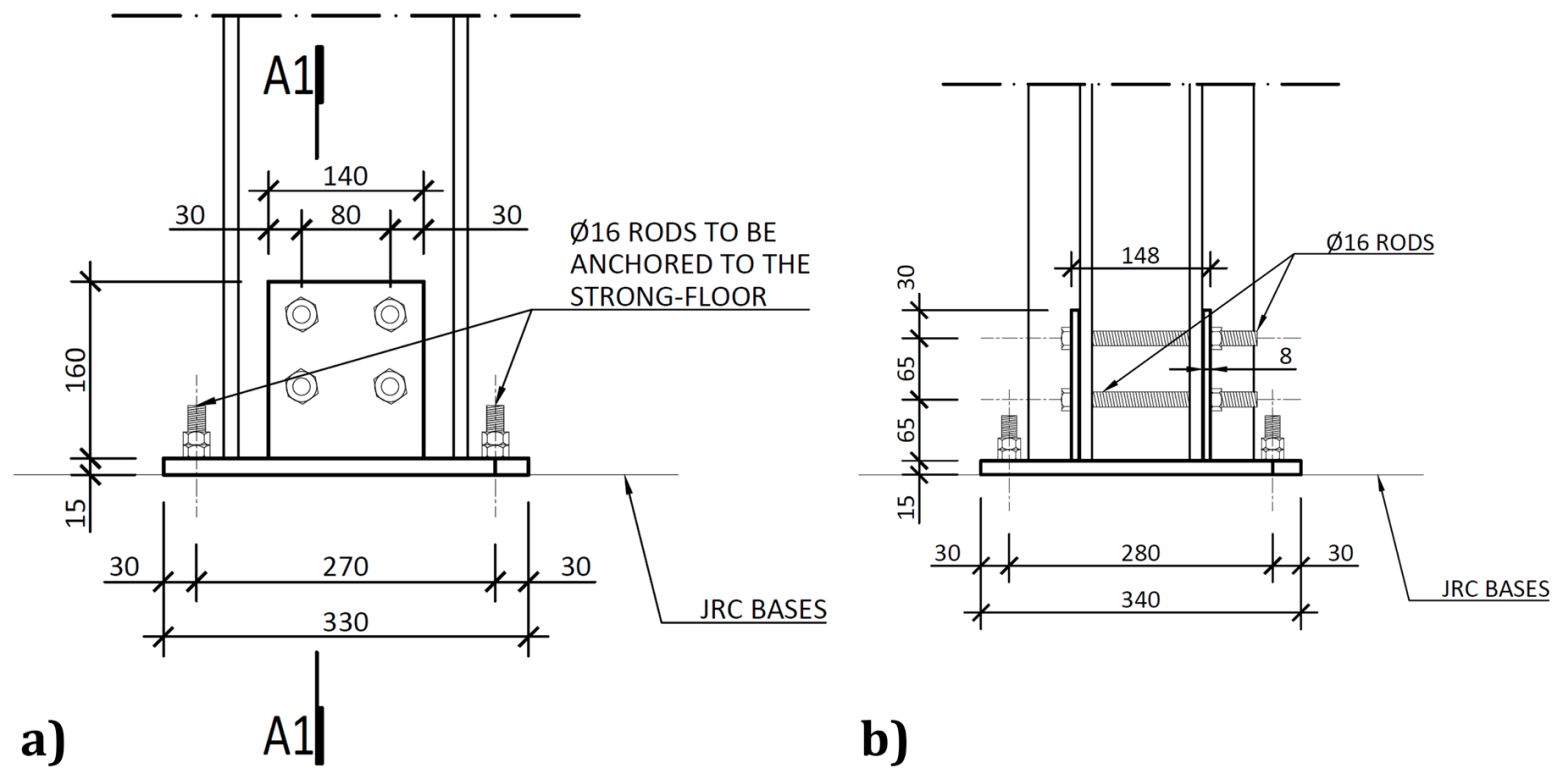
Column-to-footing connections: **a**) detail A ( *cf*. Figure 2); and **b**) section A1-A1 (dimensions in mm).

As mentioned, the column-to-footing connections, depicted in Figure 6, comprised double-gusset plates welded to a base plate.

**Figure 7.**
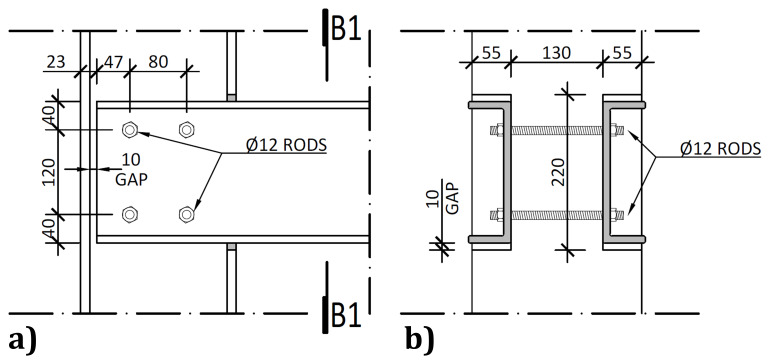
Longitudinal beam to column connections: **a**) external side view; and **b**) section B1-B1 (dimensions in mm).

The connections between the columns and the longitudinal beams did not comprise auxiliary parts, with both members being directly bolted to each other. To that end, at the beam and column intersections, the flanges of the columns were locally cut, as depicted in Figure 7, leaving a 10 mm gap between the flanges of the columns and the flanges of the webs.

**Figure 8.**
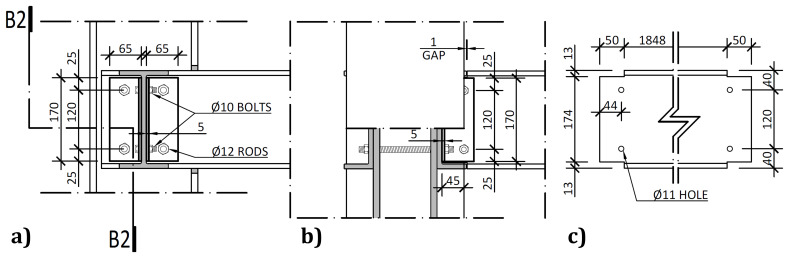
Transverse beam-to-column connections: **a**) internal side view; **b**) section B2-B2; and **c**) geometrical details of transverse beams (dimensions in mm).

On the other hand, the connections between the columns and the transverse beam, detailed in Figure 8, comprised stainless steel web cleats.

**Figure 9.**
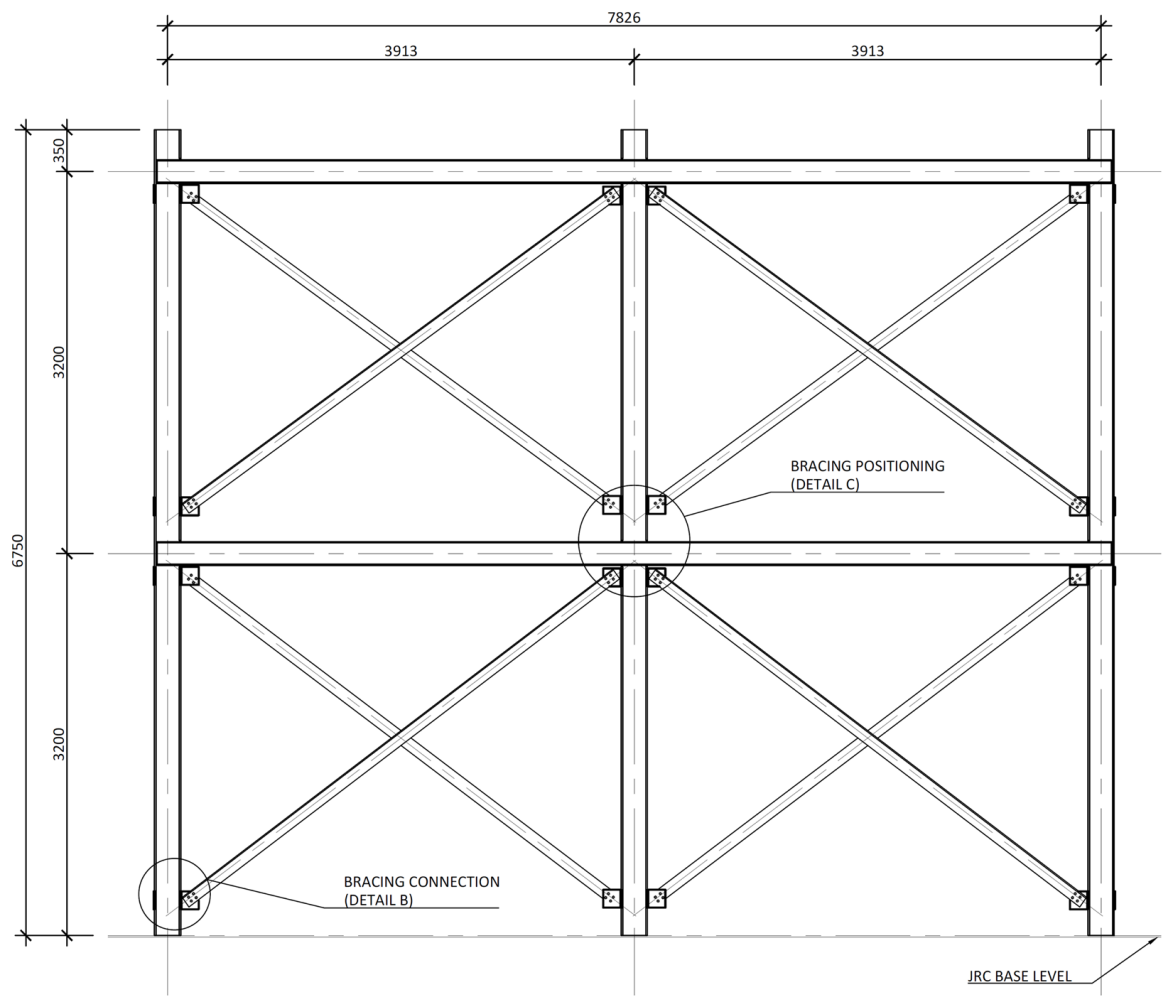
Elevation view of braced specimens, without slabs (dimensions in mm).

**Figure 10.**
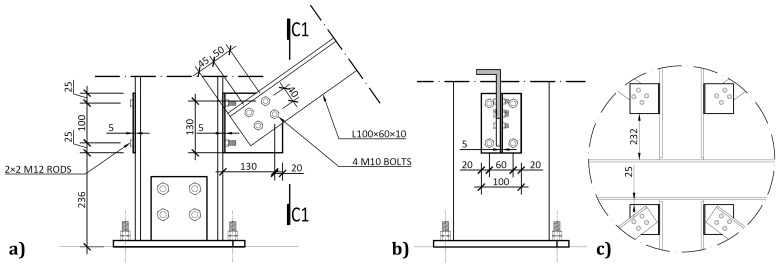
Bracing-to-column connections: **a**) detail B ( *cf*. Figure 9); **b**) section C1-C1; and **c**) Detail C (dimensions in mm).

The bracing system installed in specimens BFrame-M and BFrame-C, illustrated in Figure 9, comprised L100 profiles ( *cf*. Figure 1) connected to the columns, using metallic auxiliary parts, comprising a gusset plate welded to a base plate. Figure 10 presents the details of these connections.

**Figure 11.**
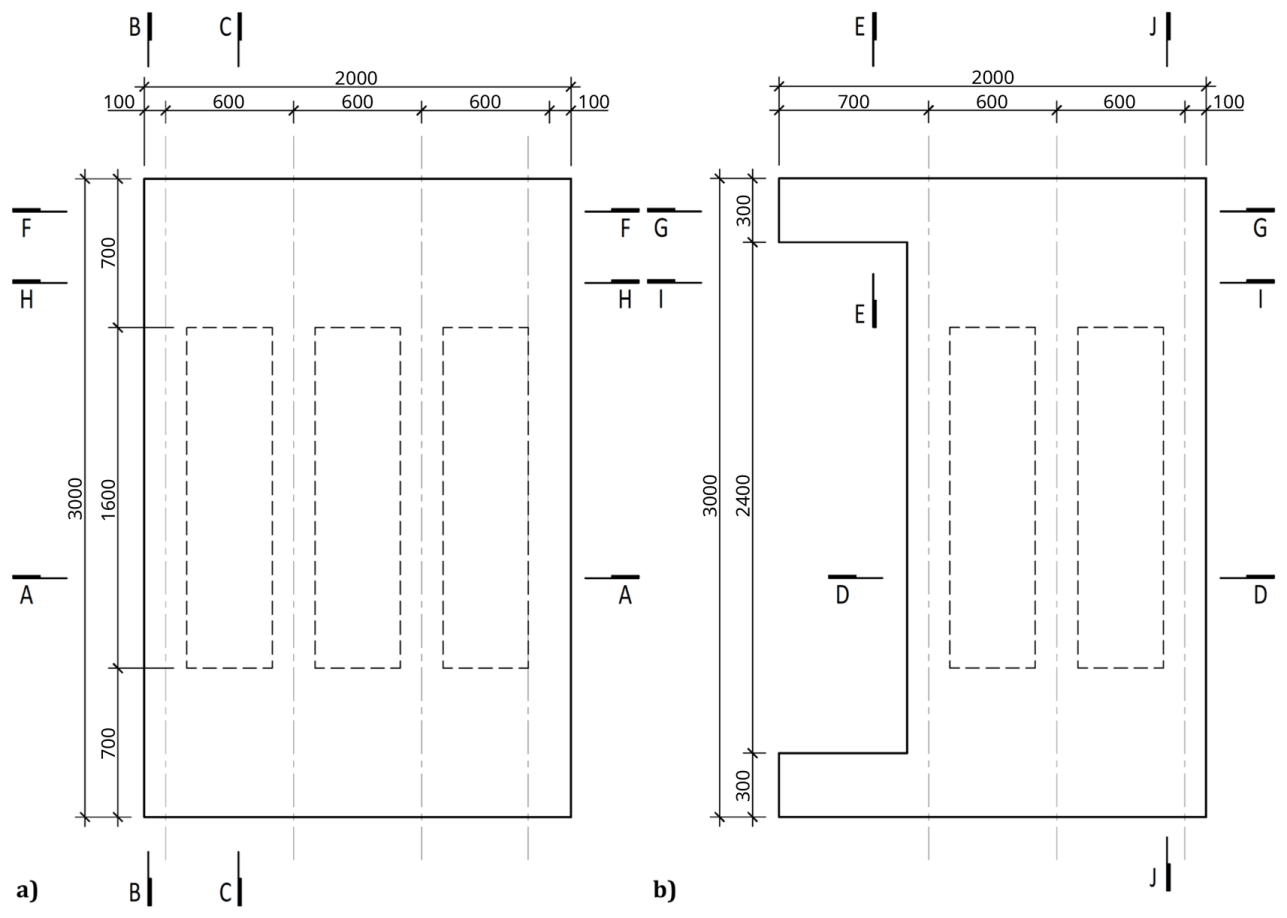
Floor slab parts: **a**) interior part; **b**) exterior part (dimensions in mm).

Regarding the concrete slabs, as mentioned, they were not a part of the structural system under testing, but a part of the test setup, simulating the vertical loads, and allowing to introduce the lateral loads. In this context, each slab floor was divided in four precast modules, two interior ones and two lateral ones, shown in Figures 11a and 11b, respectively. The design of the slabs guaranteed that there was no concrete-GFRP composite action in the vicinity of the supports, and that the slabs did not transmit bending moments from one span to the other.

**Figure 12.**
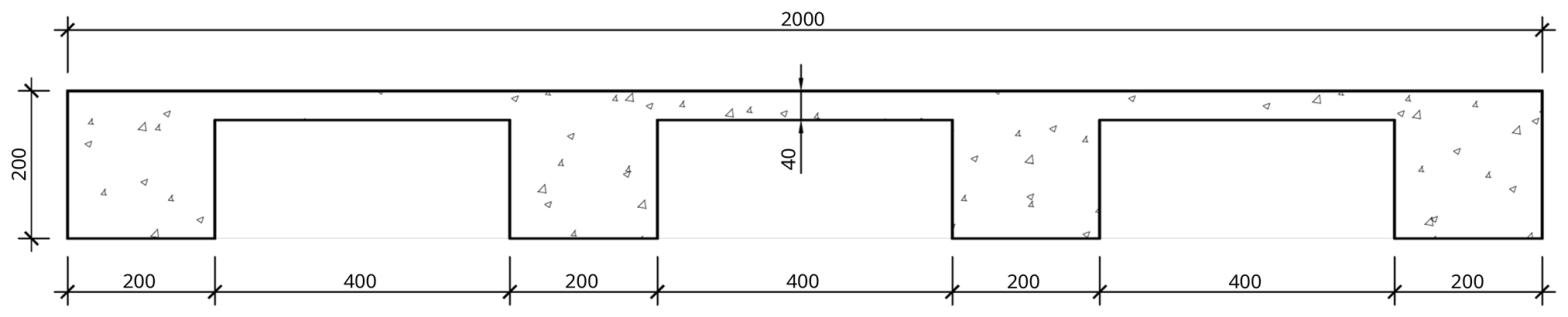
Floor slabs: section A-A, *cf*. Figure 11 (dimensions in mm).

**Figure 13.**
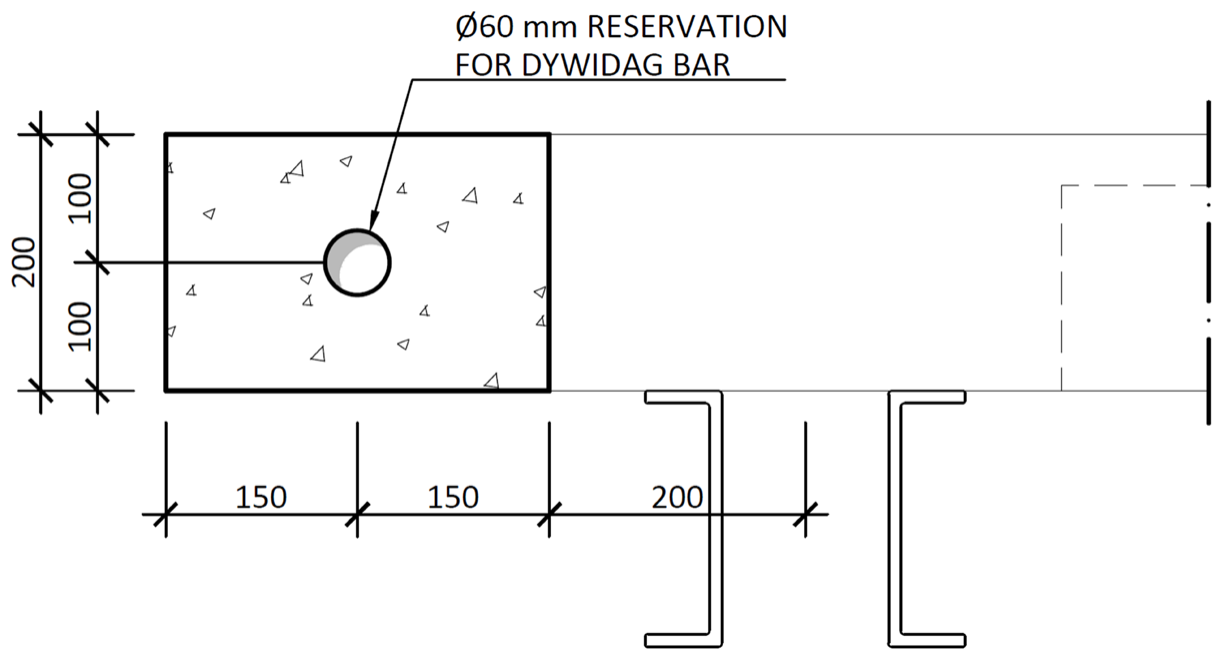
Floor slabs: section E-E, *cf*. Figure 11 (dimensions in mm).

The nominal overall thickness of the slabs was 200 mm, and expanded polystyrene blocks were used as lost formwork to decrease the overall weight of the slabs, as shown in Figure 12. Moreover, slab overhangs, depicted in Figure 13, were provided on both sides of the frame, which also extended longitudinally beyond the GFRP frame. The actuators applied the horizontal loads to these overhangs. Reservations with 60 mm diameter were installed in the overhangs, allowing the installation of *Dywidag* bars used to reverse the loading during the cyclic tests. Overall, the nominal mass of the interior slabs was 2275 kg, while the exterior slabs add a nominal mass of 1802 kg.

**Figure 14.**
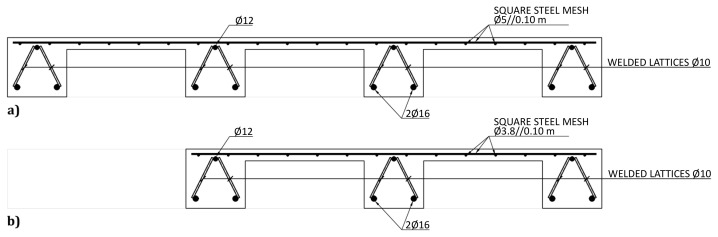
Reinforcement detailing of slabs: **a**) section A-A and **b**) section D-D ( *cf*. Figure 11).

The slabs were internally reinforced with steel rebars. Figure 14 to Figure 16 show the reinforcement detailing according to the sections identified in Figure 11.

**Figure 15.**
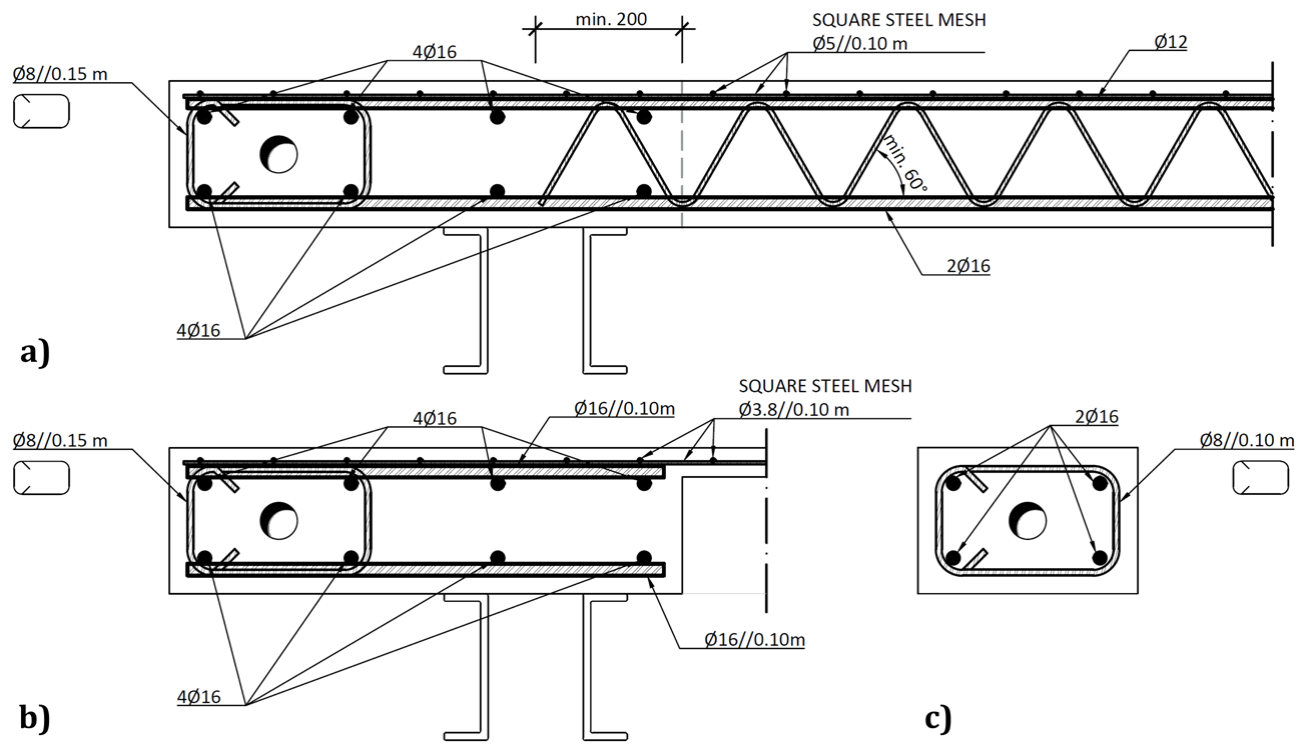
Reinforcement detailing of slabs: **a**) section B-B, **b**) section C-C and **c**) section E-E ( *cf*. Figure 11).

**Figure 16.**
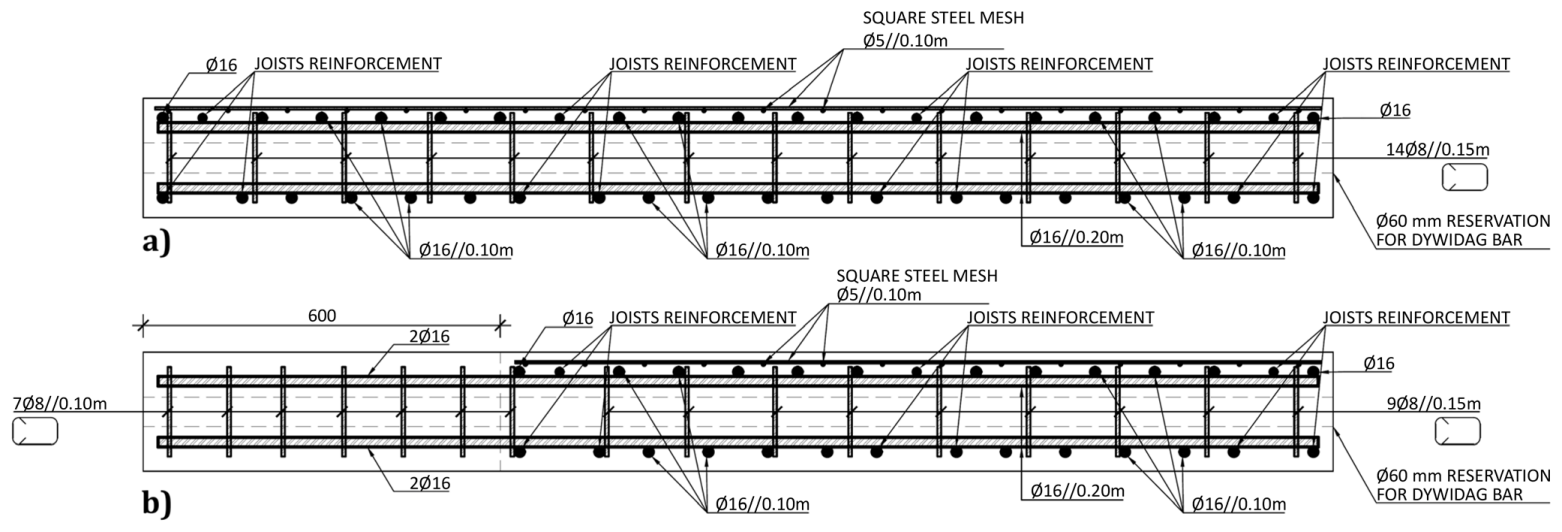
Reinforcement detailing of slabs: **a**) section F-F, and **b**) section G-G ( *cf*. Figure 11).

**Figure 17.**
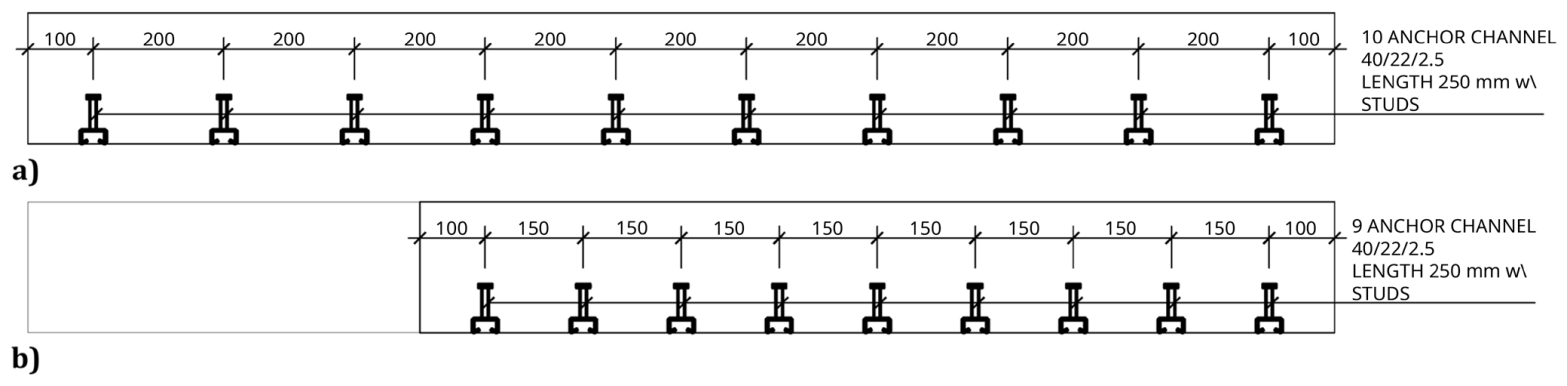
Shear connection detailing: **a**) section H-H, and **b**) section I-I, *cf*. Figure 11 (dimensions in mm).

**Figure 18.**
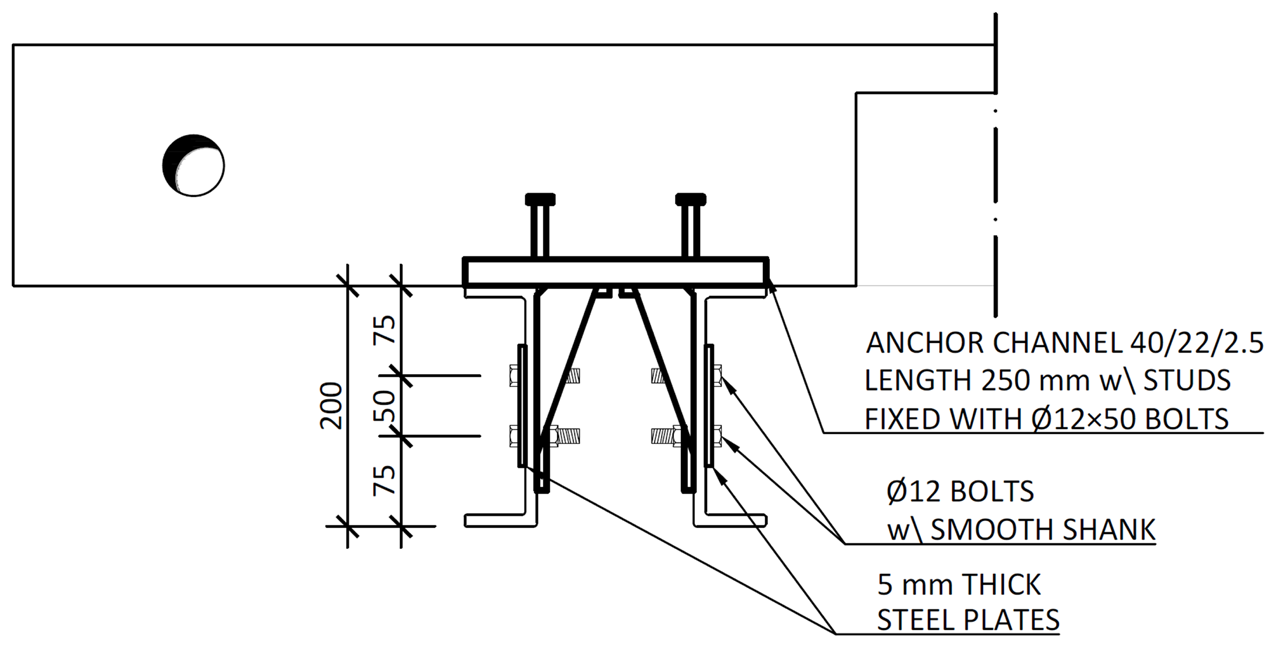
Shear connection detailing: section C-C, *cf*. Figure 11 (dimensions in mm).

The slab-beam shear connections comprised *TECNOGRIP* anchors ^18^, a system developed for precast concrete structures. To that end, anchor channel 40/22/2.5 ^18^ metallic elements were installed in the slabs, during the casting, as shown in Figure 17. After the positioning of the slabs over the frame structure, the shear connection was completed by attaching a companion steel part to the anchor channels, which were then bolted to the beams, as shown in Figure 18.

**Figure 19.**
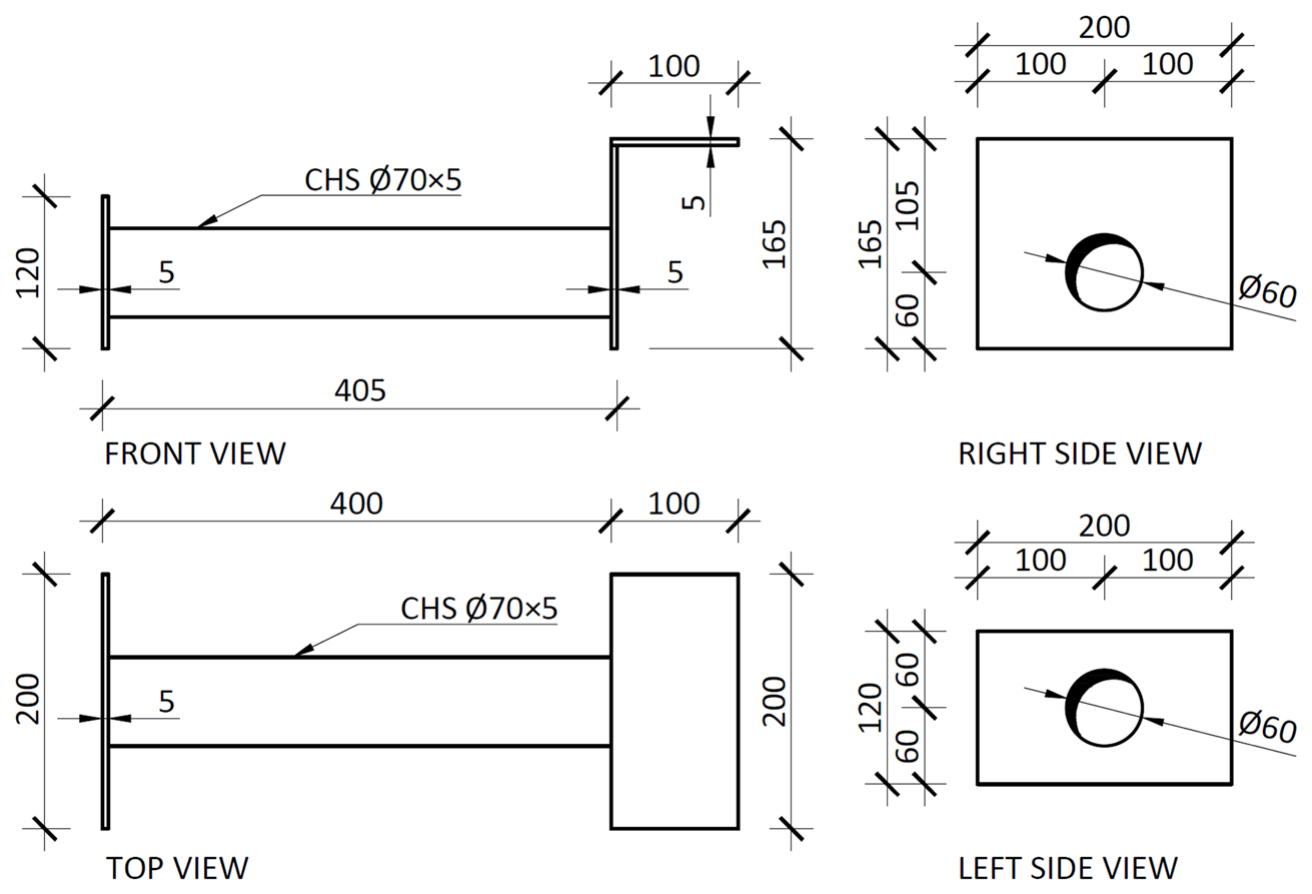
Slab connector (dimensions in mm).

Finally, the slab connector depicted in Figure 5 comprised a steel (grade S235) CHS tube, to be aligned with the slabs reservations, allowing the installation of the *dywidag* bars, welded to top plates. Figure 19 presents the details of these elements, which allowed for the prestressing of the *dywidag* bars, required to reverse the loading in the cyclic tests.

***Construction phases.*** The construction of the specimens began with the preparation of the individual structural members in MM's plant. This included the cutting to size of beam, column and bracing members, the drilling of the holes for the beam-to-column, column-to-footing and bracing-to-column connections, and the local cutting of the columns’ flanges near the beam-to-column connections ( *cf*. Figure 7). The preparation activities also included installing base plates for the inclinometers ( *cf*. Section Set-up and instrumentation) and most of the strain gauges (some were installed later in the laboratory).

The structural members were then transported to the JRC ELSA facilities, along with the metallic connection parts and the precast slabs, which were outsourced by MM.

**Figure 20.**
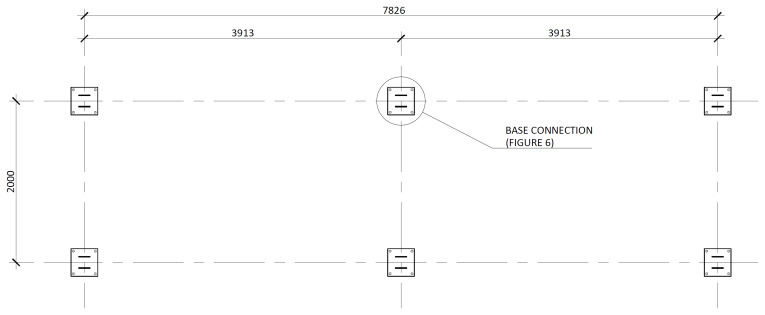
Installation of column-to-footing connection plates (dimensions in mm).

**Figure 21.**
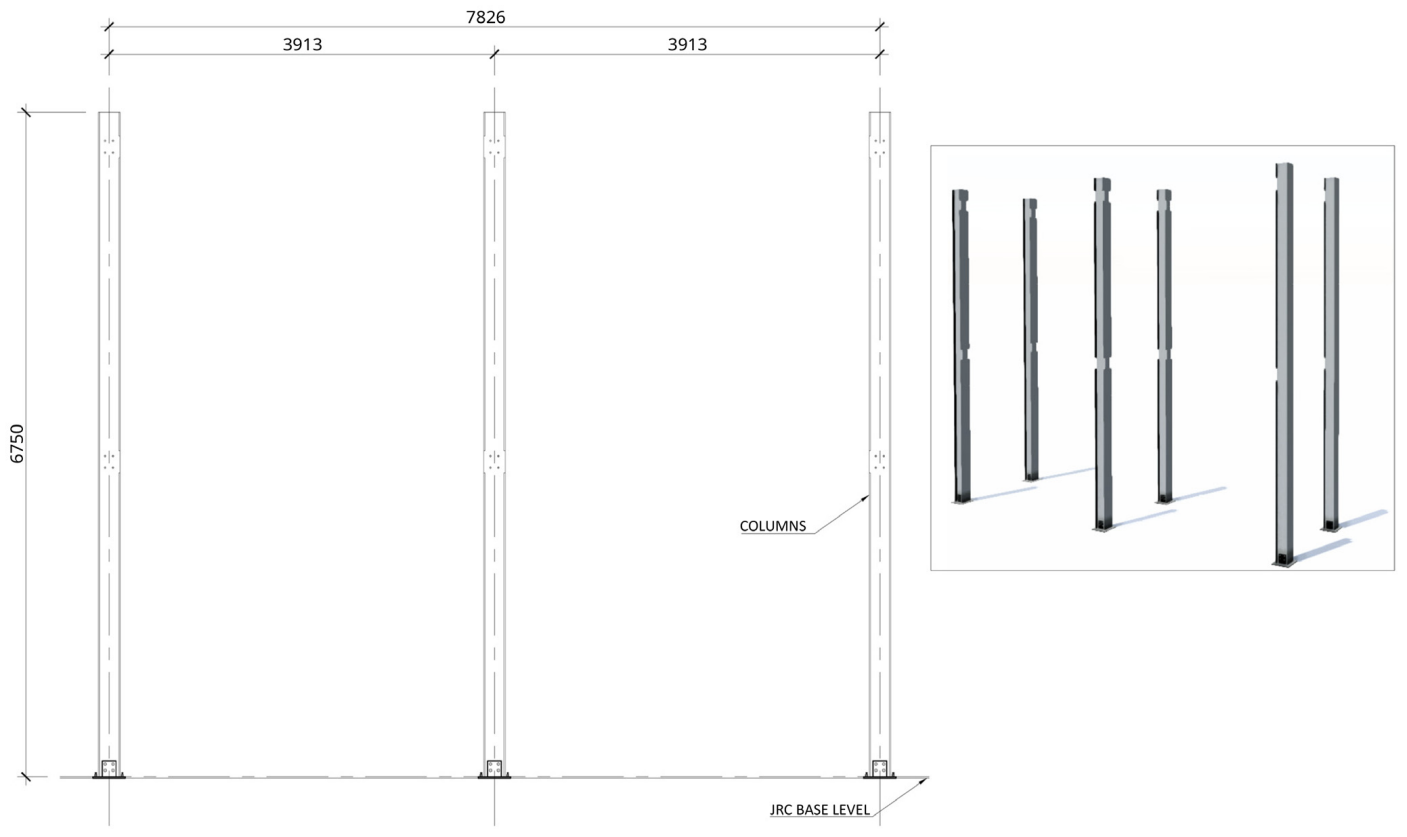
Installation of columns (dimensions in mm).

**Figure 22.**
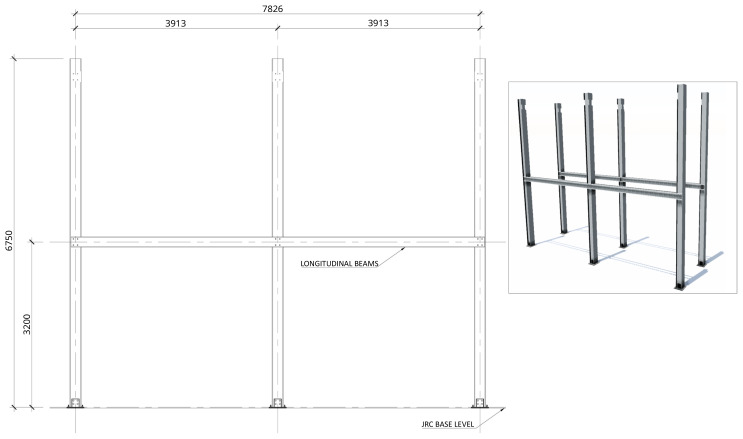
Installation of 1 ^st^ storey longitudinal beams (dimensions in mm).

**Figure 23.**
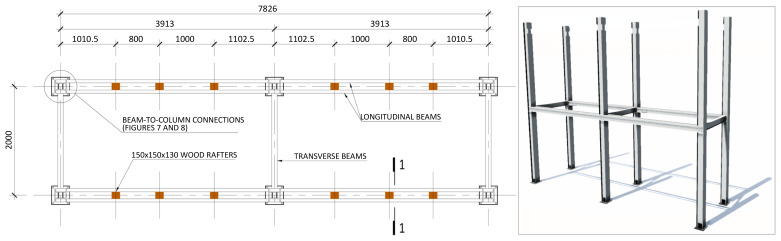
Installation of 1 ^st^ storey transverse beams and wood rafters (dimensions in mm).

**Figure 24.**
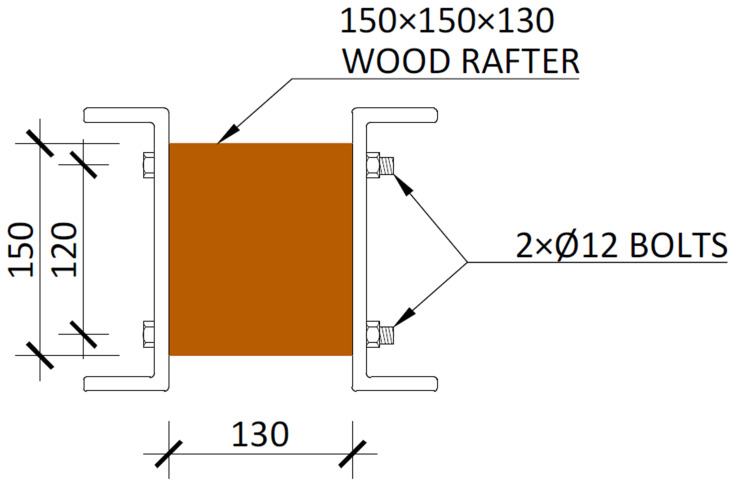
Detail of the wood rafters installed in beams (dimensions in mm).

**Figure 25.**
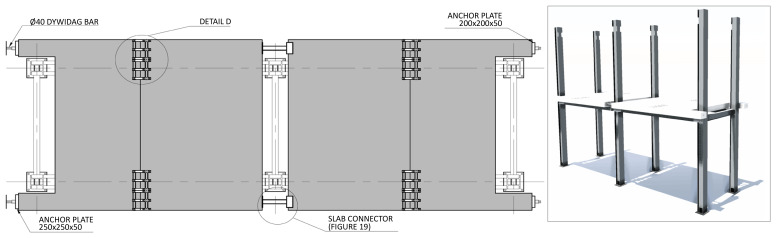
Placing of 1 ^st^ storey slabs, installation of connecting plates and slab connectors (dimensions in mm).

**Figure 26.**
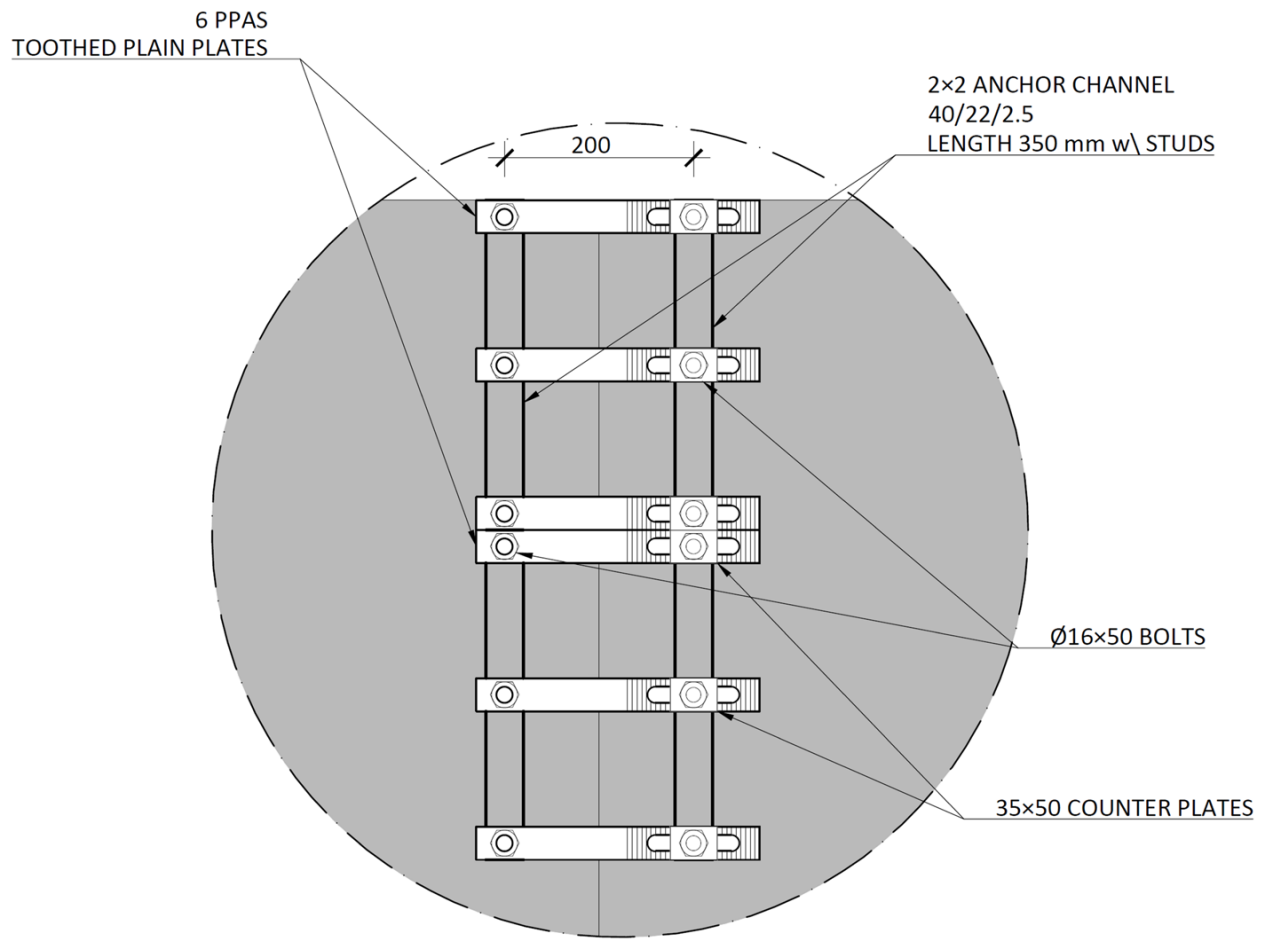
Connecting plates – Detail D (dimensions in mm).

**Figure 27.**
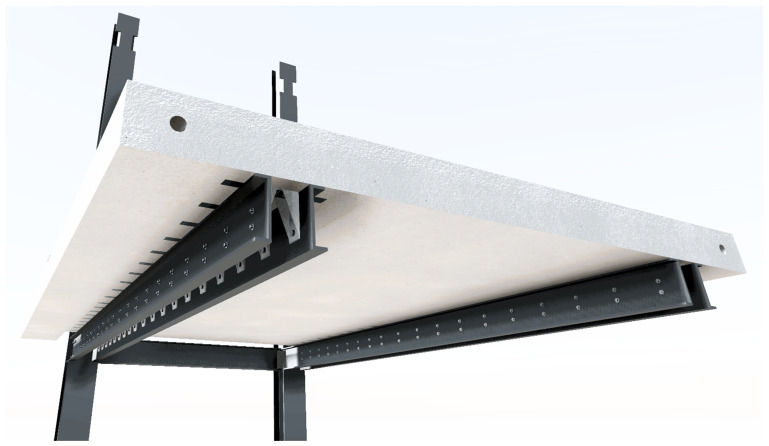
Illustration of the shear connectors configuration on the 1 ^st^ storey slabs.

**Figure 28.**
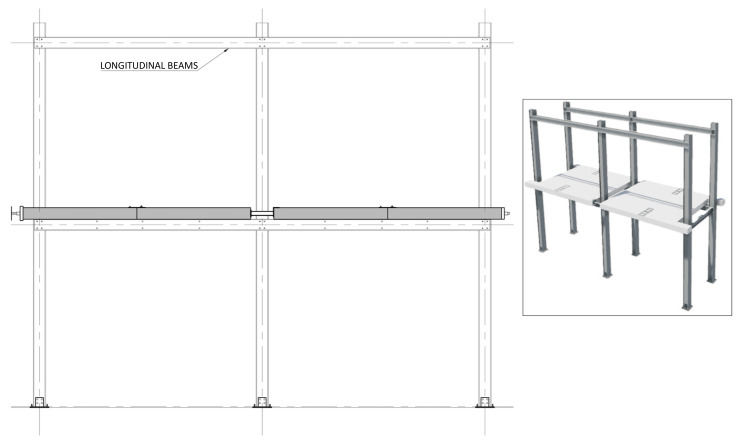
Installation of 2 ^nd^ storey longitudinal beams.

**Figure 29.**
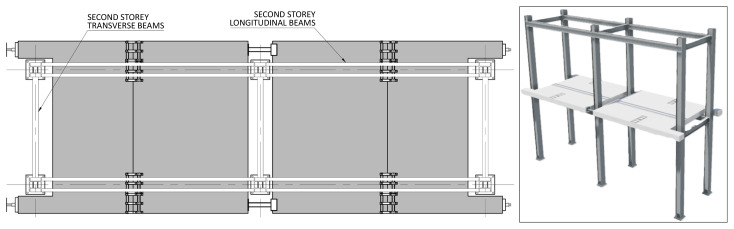
Installation of 2 ^nd^ storey transverse beams.

**Figure 30.**
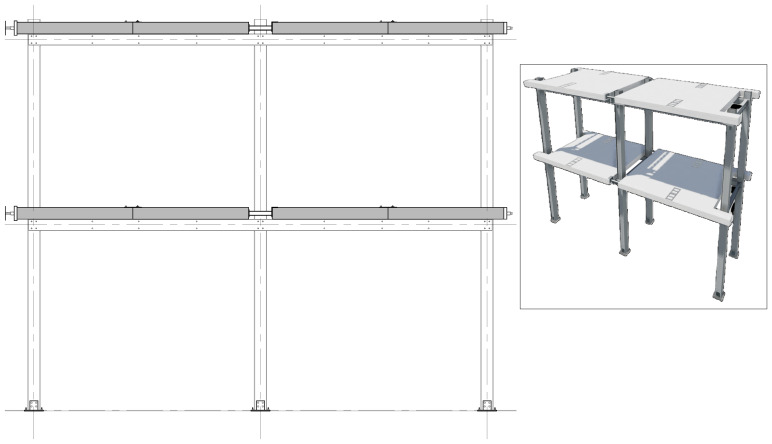
Placing of 2 ^nd^ storey slabs, installation of connecting plates and slab connectors.

**Figure 31.**
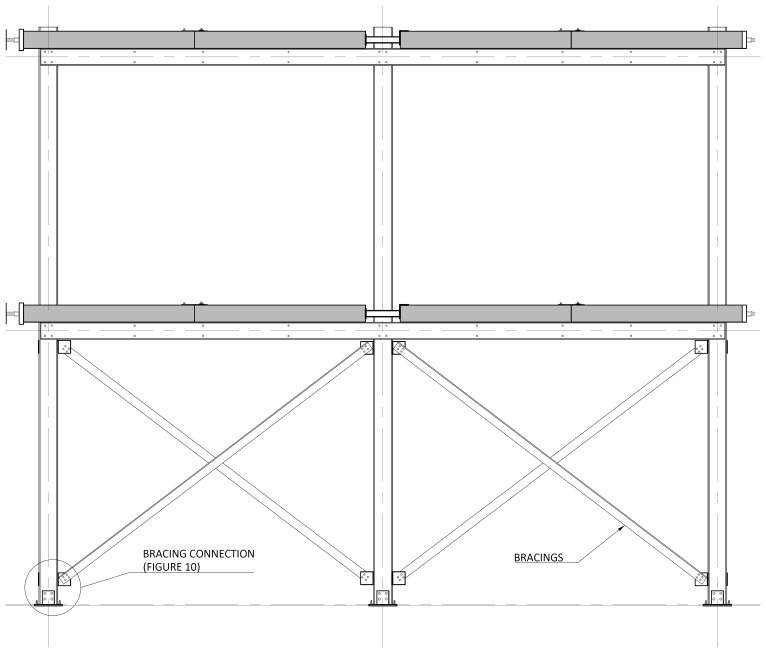
Installation of the bottom bracings (only for BFrame-M and BFrame-C).

**Figure 32.**
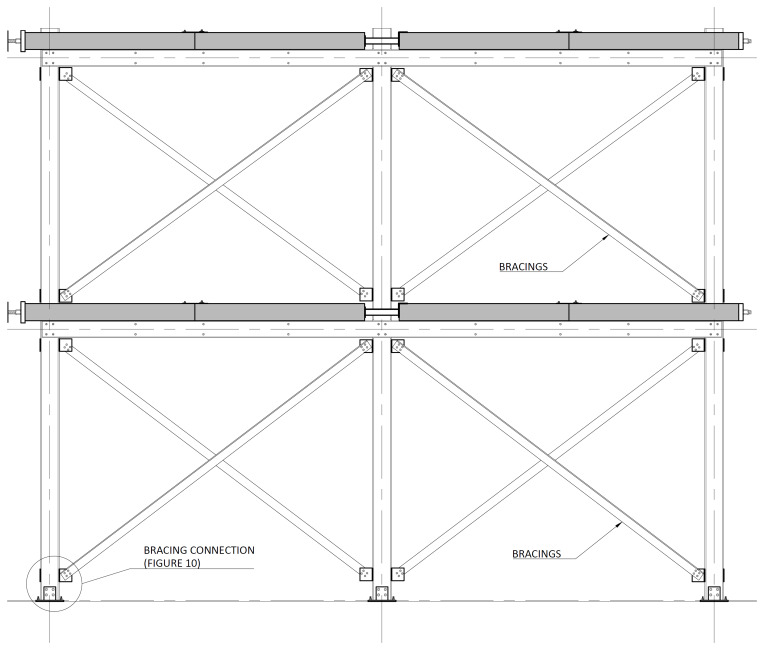
Installation of the top bracings (only for BFrame-M and BFrame-C).

**Figure 33.**
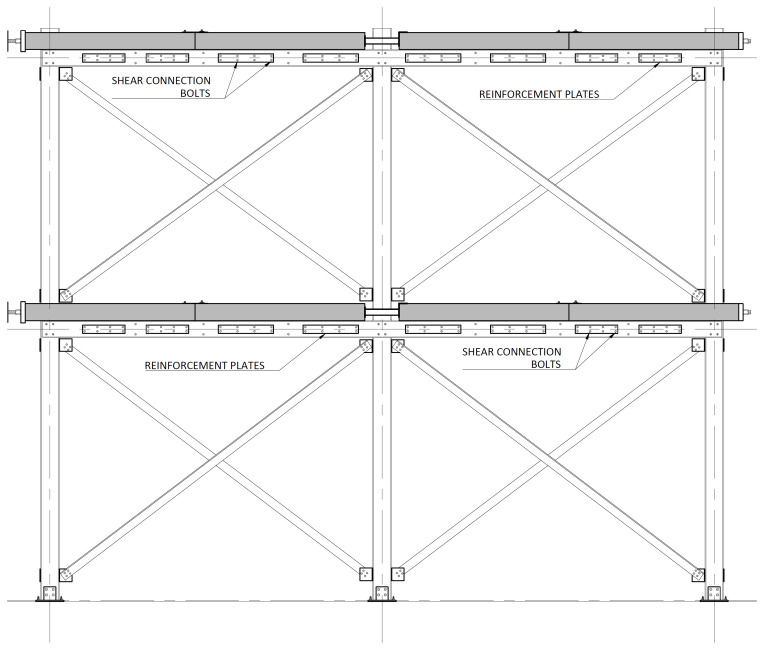
Installation of the shear connectors in the beams (bracings only for BFrame-M and BFrame-C).

At the ELSA facilities, the specimen construction process was as follows:

1. Attachment of the base plate double gussets to the base plates (already anchored to the floor, Figure 20);
2. Positioning of the columns and bolting to the base plates ( Figure 21);
3. Installation of the longitudinal beams in the 1 ^st^ storey ( Figure 22);
4. Installation of the transverse beams in the 1 ^st^ storey ( Figure 23);
5. Installation of wood rafters at quarters of the beams span ^a^ ( Figure 23 and Figure 24);
6. Placement of the precast slabs over the 1 ^st^ storey beams, including the *dywidag* bars, connecting steel plates and slab connectors ( Figure 25 and Figure 26);
7. Installation of the shear connectors ( *cf*. Figure 18) in the 1 ^st^ storey slabs, without fastening to the beams ( Figure 27);
8. Installation of the longitudinal beams in the 2 ^nd^ storey ( Figure 28);
9. Installation of the transverse beams in the 2 ^nd^ storey ( Figure 29);
10. Installation of wood rafters at quarters of the beams span in the 2 ^nd^ storey;
11. Placement of the precast slabs over the 2 ^nd^ storey beams, including the *Dywidag* bars, connecting steel plates and slab connectors ( Figure 30);
12. Installation of the shear connectors ( *cf*. Figure 18) in the 2 ^nd^ storey slabs, without fastening to the beams;
13. Application of prestress to the *Dywidag* bars;
14. Installation of the bottom bracings ( Figure 31, specimens BFrame-M and BFrame-C only);
15. Installation of the top bracings ( Figure 32, specimens BFrame-M and BFrame-C only);
16. Fastening of the shear connectors to the beams ( *cf*. Figure 18 and Figure 27), including drilling of the holes in the beams, and installation of the external steel strengthening plates ( Figure 33, not used in specimen uFrame-M).

It should be mentioned that for the first specimen, uFrame-M, step 16 was performed before step 13. This led to the occurrence of some damage on the shear connection system, namely the ovalisation of the beams bolt holes, which was not detected before the tests due to the presence of the washers. As a consequence, as described ahead in this paper, that particular specimen presented an asymmetric response from the onset of the test, also exhibiting sliding between the slabs and the frame. Owing to this unwanted behaviour, in the remaining specimens, the sequence of the mounting operation was modified according to what was previously described.

### Set-up and instrumentation

**Figure 34.**
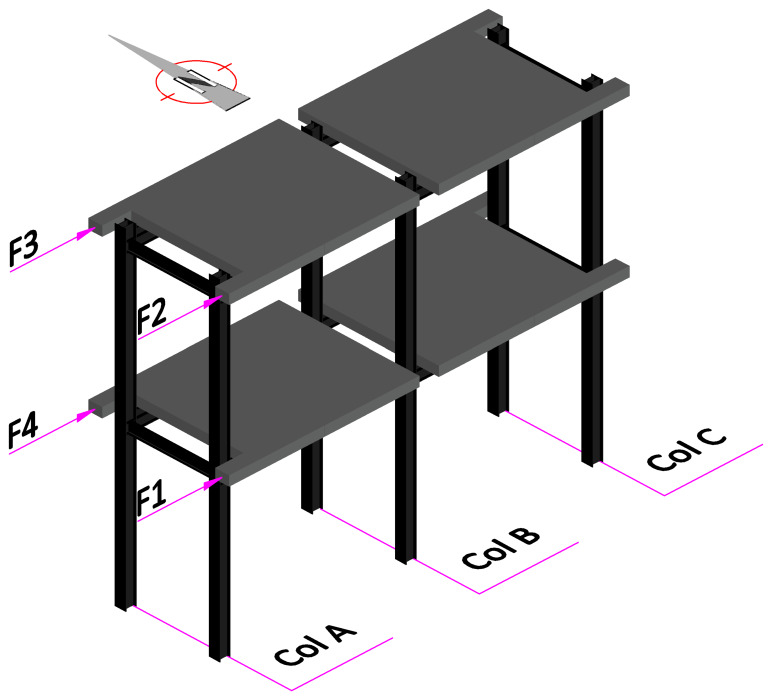
Tri-dimensional schematic view of specimens, including orientation and load and column numbering.

As mentioned, the experimental campaign focused on sway tests of large-scale GFRP frame structures. Figure 34 presents a scheme of the test setup, showing the nomenclature of the load application points and of the columns. The reaction wall was located on the West side of the specimens. In the opposite side ( *i.e*., the East side), the horizontal displacements were measured at both the columns and the slabs. The loads were applied by 4 actuators, with a capacity of 500 kN and total stroke of 500 mm. The loads were measured by load cells built-in the actuators ( *Maywood* 500 kN). The horizontal displacements were also measured by the built-in digital sensors of the actuators ( *Temposonics*) positioned on the West side of the specimens. Additionally, 8 sensors (linear encoders, *Heidenhain MSA 375*) on the East side of the specimens were used to measure the displacements of the concrete slabs and the composite columns. This also allowed evaluating possible slip deformations due to the potential flexibility of the shear connections. Please note that the calibration reports of force and displacement sensors are included in the dataset. For additional details about the interpretation of the calibration reports, the reader can refer to the Technical Report No JRC139347 ^19^. Figure 34 shows a schematic tri-dimensional view of the specimens, indicating their position with respect to the compass, also showing the numbering of the load measuring equipment and the nomenclature of the columns. Regarding the nomenclature of the displacement measurements, the slab displacements on the West side are identified by Con, while, on the East side, the slab and column displacements are identified as HS and HC, respectively. The numbering of the displacement transducers follows the same distribution adopted for the applied loads ( *cf*. Figure 34).

**Figure 35.**
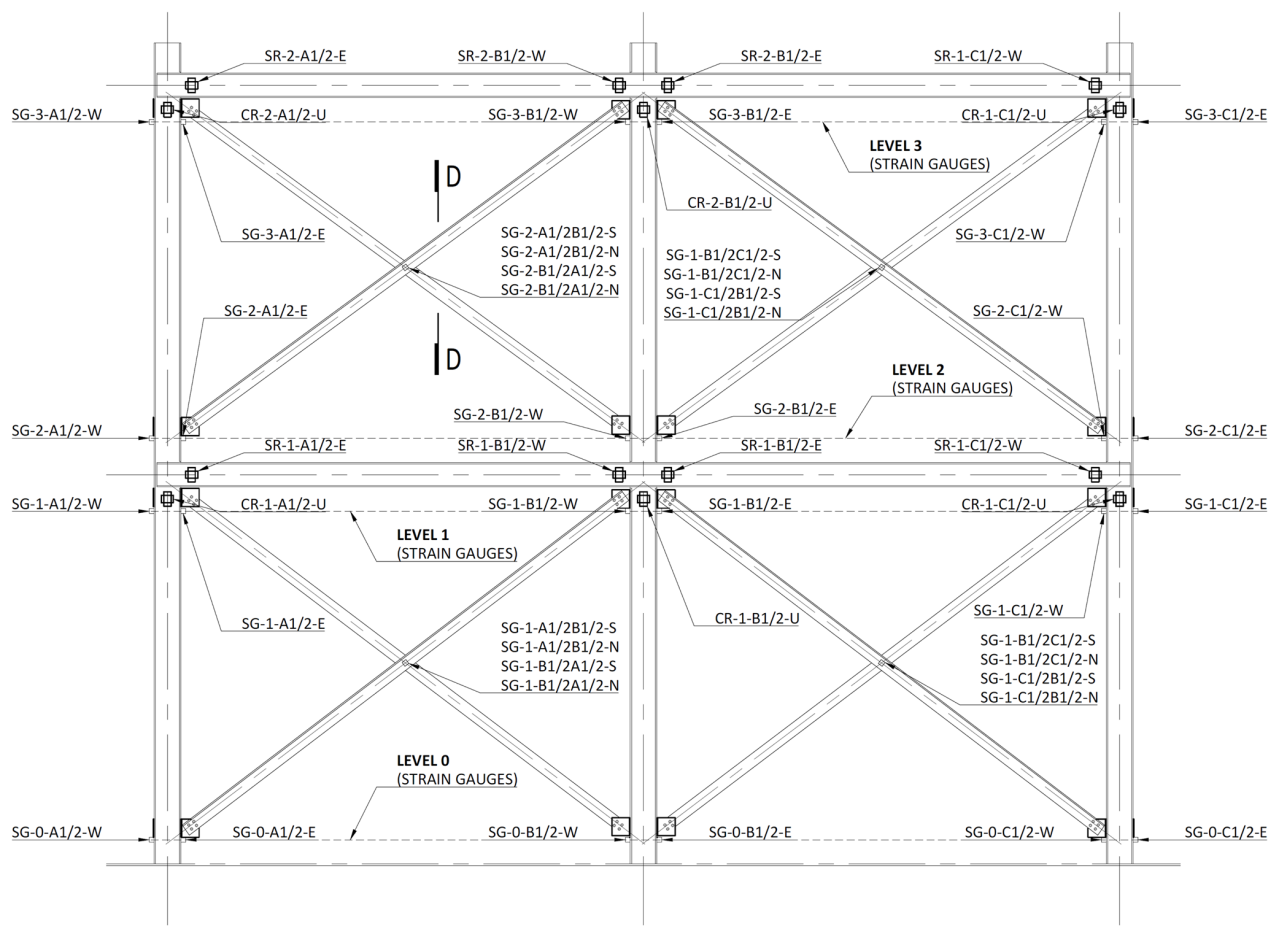
Positioning of the inclinometers and strain gauges in South (1) and North (2) lateral frames.

The rotations of the columns and beams were measured in the vicinity of the beam-to-column connections, with inclinometers from *SEIKA* (model *SBU1*), with stroke of ±5°, installed as depicted in Figure 35. The column inclinometers were positioned under the longitudinal beams, at ~ 200 mm from their mid-axis, and named *CR-Level-Column-U*, where *Level* refers to the storey (1 or 2) and the *Column* also identifies the longitudinal lateral frame, *i.e*., A1 refers to column A ( *cf*. Figure 34) of the South lateral frame (1), while A2 refers to column A of the North lateral frame (2). U simply highlights that the inclinometer is placed under the beam. As an example, CR-2-B1-U refers to the inclinometer placed in column B of the South lateral frame under the 2 ^nd^ storey beam. Similarly, the beam inclinometers were named *SR-Level-Column-Side*, where *Level* refers to the storey (1 or 2), *Column* includes the identification of the lateral frame and, in this case, *Side* indicates if the inclinometer is positioned in the West (W) or East (E) side of the column. For instance, SR-1-B2-W refers to the inclinometer positioned at the West side of the column B of the North frame’s 1 ^st^ storey. It should be mentioned that the beam inclinometers were positioned at ~200 mm from the vertical axis of the columns.

Figure 35 also depicts the positions of the strain measurement points on the columns and on the bracings. Electric strain gauges from TML, models FLKB-6-11-3LJC-F or FLA-6-11 were used. For the columns, two strain gauges were used per section, positioned at the mid-width of both flanges. The measurements were made at 4 levels: at distances of ~200 mm (0), ~2900 mm (1), ~3500 mm (2), and 6100 mm (3) from the bottom of the column profiles. The measuring points were named *SG-Level-Column-Side*, in which the *Column* also includes the indication of the lateral frame (1 for South and 2 for North), while the *Side* indicates the strain gauge bonded on the West (W) or East (E) flange of the respective column.

**Figure 36.**
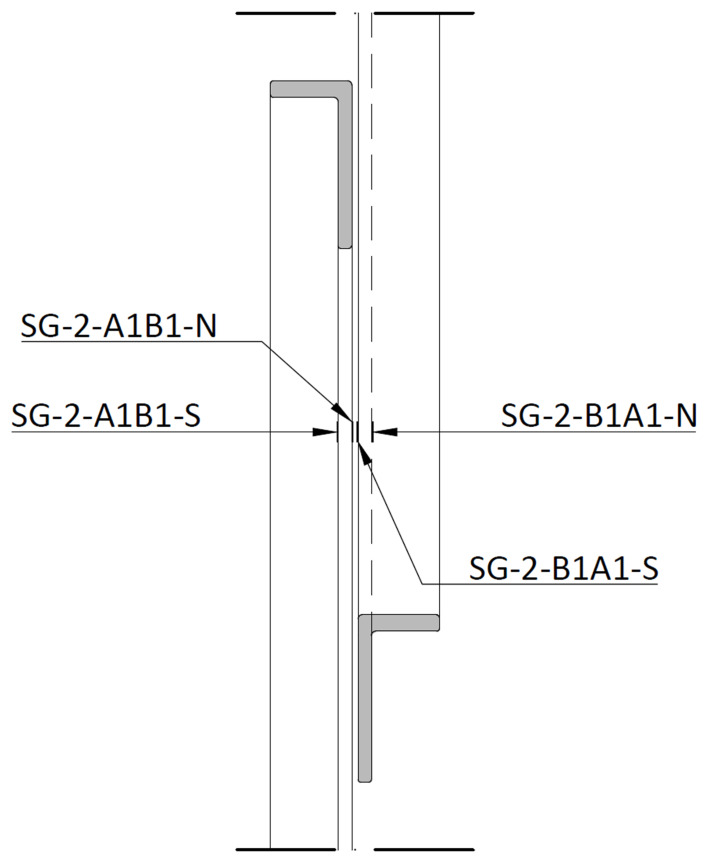
Section D-D ( *cf*. Figure 35): positioning of bracing strain gauges at South (1) frame.

For the bracings, the strain gauges were installed in the vicinity of mid-span, on both sides of the longer angle leg, as exemplified in Figure 35 and Figure 36. The measuring positions were named *SF-Level-Bottom column/Top column-Side*. In this case, the *Level* refers to the bracings of the first (1) or second (2) storey, respectively. *Bottom column* refers to the column to which the bracing has its lower connection, while the *Top column* identifies the column to which it has its upper connection, while *Side* refers to the position of the strain gauge in the bracings section (South or North, *cf*. Figure 36). As an example, SG-1-C2B2-N refers to the strain gauge positioned at the bottom storey (1) of the North lateral frame (2), connecting the bottom of column C2 to the top of column B2, on the North side.

Table 3 and Table 4 present the complete list of the measurements made, including the type of measurement, the position, and the specimens for which measurements were taken.

**Table 3.** List of load, displacement and rotation measurements.

Type	Nomenclature	Frame	Level	Position	Specimens
Load	F1	South	1 ^st^ storey	Slab, near A1	All
	F2	South	2 ^nd^ storey	Slab, near A1	All
	F3	North	1 ^st^ storey	Slab, near A2	All
	F4	North	2 ^nd^ storey	Slab, near A2	All
Displacement	Con1	South	1 ^st^ storey	Slab, near A1	All
	Con2	South	2 ^nd^ storey	Slab, near A1	All
	Con3	North	1 ^st^ storey	Slab, near A2	All
	Con4	North	2 ^nd^ storey	Slab, near A2	All
	HS1	South	1 ^st^ storey	Slab, near C1	All
	HS2	South	2 ^nd^ storey	Slab, near C1	All
	HS3	North	1 ^st^ storey	Slab, near C2	All
	HS4	North	2 ^nd^ storey	Slab, near C2	All
	HC1	South	1 ^st^ storey	C1	All
	HC2	South	2 ^nd^ storey	C1	All
	HC3	North	1 ^st^ storey	C2	All
	HC4	North	2 ^nd^ storey	C2	All
Rotation	CR-1-A1-U	South	1 ^st^ storey	A1	All
	SR-1-A1-E	South	1 ^st^ storey	Beam, East of A1	All
	CR-1-B1-U	South	1 ^st^ storey	B1	All
	SR-1-B1-W	South	1 ^st^ storey	Beam, West of B1	All
	SR-1-B1-E	South	1 ^st^ storey	Beam, East of B1	All
	CR-1-C1-U	South	1 ^st^ storey	C1	All
	SR-1-C1-W	South	1 ^st^ storey	Beam, West of C1	All
	CR-2-A1-U	South	2 ^nd^ storey	A1	All
	SR-2-A1-E	South	2 ^nd^ storey	Beam, East of A1	All
	CR-2-B1-U	South	2 ^nd^ storey	B1	All
	SR-2-B1-W	South	2 ^nd^ storey	Beam, West of B1	All
	SR-2-B1-E	South	2 ^nd^ storey	Beam, East of B1	All
	CR-2-C1-U	South	2 ^nd^ storey	C1	All
	SR-2-C1-W	South	2 ^nd^ storey	Beam, West of C1	All
	CR-1-A2-U	North	1 ^st^ storey	A2	All
	SR-1-A2-E	North	1 ^st^ storey	Beam, East of A2	All
	CR-1-B2-U	North	1 ^st^ storey	B2	All
	SR-1-B2-W	North	1 ^st^ storey	Beam, West of B2	All
	SR-1-B2-E	North	1 ^st^ storey	Beam, East of B2	All
	CR-1-C2-U	North	1 ^st^ storey	C2	All
	SR-1-C2-W	North	1 ^st^ storey	Beam, West of C2	All
	CR-2-A2-U	North	2 ^nd^ storey	A2	All
	SR-2-A2-E	North	2 ^nd^ storey	Beam, East of A2	All
	CR-2-B2-U	North	2 ^nd^ storey	B2	All
	SR-2-B2-W	North	2 ^nd^ storey	Beam, West of B2	All
	SR-2-B2-E	North	2 ^nd^ storey	Beam, East of B2	All
	CR-2-C2-U	North	2 ^nd^ storey	C2	All
	SR-2-C2-W	North	2 ^nd^ storey	Beam, West of C2	All

**Table 4.** List of strain measurements.

Type	Nomenclature	Frame	Level	Position	Specimens
Strains	SG-0-A1-W	South	Level 0	A1, West	All
	SG-0-A1-E	South	Level 0	A1, East	All
	SG-1-A1-W	South	Level 1	A1, West	All
	SG-1-A1-E	South	Level 1	A1, East	All
	SG-2-A1-W	South	Level 2	A1, West	All, except uFrame-M
	SG-2-A1-E	South	Level 2	A1, East	All, except uFrame-M
	SG-3-A1-W	South	Level 3	A1, West	All
	SG-3-A1-E	South	Level 3	A1, East	All
	SG-0-B1-W	South	Level 0	B1, West	All
	SG-0-B1-E	South	Level 0	B1, East	All
	SG-1-B1-W	South	Level 1	B1, West	All
	SG-1-B1-E	South	Level 1	B1, East	All
	SG-2-B1-W	South	Level 2	B1, West	All, except uFrame-M
	SG-2-B1-E	South	Level 2	B1, East	All, except uFrame-M
	SG-3-B1-W	South	Level 3	B1, West	All
	SG-3-B1-E	South	Level 3	B1, East	All
	SG-0-C1-W	South	Level 0	C1, West	All
	SG-0-C1-E	South	Level 0	C1, East	All
	SG-1-C1-W	South	Level 1	C1, West	All
	SG-1-C1-E	South	Level 1	C1, East	All
	SG-2-C1-W	South	Level 2	C1, West	All, except uFrame-M
	SG-2-C1-E	South	Level 2	C1, East	All, except uFrame-M
	SG-3-C1-W	South	Level 3	C1, West	All
	SG-3-C1-E	South	Level 3	C1, East	All
	SG-0-A2-W	North	Level 0	A2, West	All
	SG-0-A2-E	North	Level 0	A2, East	All
	SG-1-A2-W	North	Level 1	A2, West	All
	SG-1-A2-E	North	Level 1	A2, East	All
	SG-2-A2-W	North	Level 2	A2, West	All
	SG-2-A2-E	North	Level 2	A2, East	All
	SG-3-A2-W	North	Level 3	A2, West	All
	SG-3-A2-E	North	Level 3	A2, East	All
	SG-0-B2-W	North	Level 0	B2, West	All
	SG-0-B2-E	North	Level 0	B2, East	All
	SG-1-B2-W	North	Level 1	B2, West	All
	SG-1-B2-E	North	Level 1	B2, East	All
	SG-2-B2-W	North	Level 2	B2, West	All
	SG-2-B2-E	North	Level 2	B2, East	All
	SG-3-B2-W	North	Level 3	B2, West	All
	SG-3-B2-E	North	Level 3	B2, East	All
	SG-0-C2-W	North	Level 0	C2, West	All
	SG-0-C2-E	North	Level 0	C2, East	All
	SG-1-C2-W	North	Level 1	C2, West	All
	SG-1-C2-E	North	Level 1	C2, East	All
	SG-2-C2-W	North	Level 2	C2, West	All
	SG-2-C2-E	North	Level 2	C2, East	All
	SG-3-C2-W	North	Level 3	C2, West	All
	SG-3-C2-E	North	Level 3	C2, East	All
	SG-1-A1B1-S	South	1 ^st^ Storey	Bracing, bottom A to top B	BFrame-M and -C
	SG-1-A1B1-N	South	1 ^st^ Storey	Bracing, bottom A to top B	BFrame-M and -C
	SG-1-B1A1-S	South	1 ^st^ Storey	Bracing, bottom B to top A	BFrame-M and -C
	SG-1-B1A1-N	South	1 ^st^ Storey	Bracing, bottom B to top A	BFrame-M and -C
	SG-1-B1C1-S	South	1 ^st^ Storey	Bracing, bottom B to top C	BFrame-M and -C
	SG-1-B1C1-N	South	1 ^st^ Storey	Bracing, bottom B to top C	BFrame-M and -C
	SG-1-C1B1-S	South	1 ^st^ Storey	Bracing, bottom C to top B	BFrame-M and -C
	SG-1-C1B1-N	South	1 ^st^ Storey	Bracing, bottom C to top B	BFrame-M and -C
	SG-1-A2B2-S	South	2 ^nd^ Storey	Bracing, bottom A to top B	BFrame-M and -C
	SG-1-A2B2-N	South	2 ^nd^ Storey	Bracing, bottom A to top B	BFrame-M and -C
	SG-1-B2A2-S	South	2 ^nd^ Storey	Bracing, bottom B to top A	BFrame-M and -C
	SG-1-B2A2-N	South	2 ^nd^ Storey	Bracing, bottom B to top A	BFrame-M and -C
	SG-1-B2C2-S	South	2 ^nd^ Storey	Bracing, bottom B to top C	BFrame-M and -C
	SG-1-B2C2-N	South	2 ^nd^ Storey	Bracing, bottom B to top C	BFrame-M and -C
	SG-1-C2B2-S	South	2 ^nd^ Storey	Bracing, bottom C to top B	BFrame-M and -C
	SG-1-C2B2-N	South	2 ^nd^ Storey	Bracing, bottom C to top B	BFrame-M and -C
	SG-2-A1B1-S	North	1 ^st^ Storey	Bracing, bottom A to top B	BFrame-M and -C
	SG-2-A1B1-N	North	1 ^st^ Storey	Bracing, bottom A to top B	BFrame-M and -C
	SG-2-B1A1-S	North	1 ^st^ Storey	Bracing, bottom B to top A	BFrame-M and -C
	SG-2-B1A1-N	North	1 ^st^ Storey	Bracing, bottom B to top A	BFrame-M and -C
	SG-2-B1C1-S	North	1 ^st^ Storey	Bracing, bottom B to top C	BFrame-M and -C
	SG-2-B1C1-N	North	1 ^st^ Storey	Bracing, bottom B to top C	BFrame-M and -C
	SG-2-C1B1-S	North	1 ^st^ Storey	Bracing, bottom C to top B	BFrame-M and -C
	SG-2-C1B1-N	North	1 ^st^ Storey	Bracing, bottom C to top B	BFrame-M and -C
	SG-2-A2B2-S	North	2 ^nd^ Storey	Bracing, bottom A to top B	BFrame-M and -C
	SG-2-A2B2-N	North	2 ^nd^ Storey	Bracing, bottom A to top B	BFrame-M and -C
	SG-2-B2A2-S	North	2 ^nd^ Storey	Bracing, bottom B to top A	BFrame-M and -C
	SG-2-B2A2-N	North	2 ^nd^ Storey	Bracing, bottom B to top A	BFrame-M and -C
	SG-2-B2C2-S	North	2 ^nd^ Storey	Bracing, bottom B to top C	BFrame-M and -C
	SG-2-B2C2-N	North	2 ^nd^ Storey	Bracing, bottom B to top C	BFrame-M and -C
	SG-2-C2B2-S	North	2 ^nd^ Storey	Bracing, bottom C to top B	BFrame-M and -C
	SG-2-C2B2-N	North	2 ^nd^ Storey	Bracing, Bottom C to top B	BFrame-M and -C

During the tests, ELSAREC, the ELSA REal-time Controller (for further details, refer to 20), was used to monitor the response of the specimens: as also described in the Technical Report No JRC141264 ^19^, multiple acquisitions run in parallel. During the snap-back tests used for the dynamic identification of the specimens, the signals from the monitoring system were recorded at a rate of 1000 Hz. On the other hand, during the sway tests, the signals of the standard instruments ( *e.g*., inclinometers and strain gauges) were acquired at 10 Hz, while the signals from the control system ( *e.g*., control variables) were sampled at a variable rate. These last signals were recorded at the achievement of each record of the loading pattern, noting that the prototype-time variable common to both acquisition files can be used for synchronisation purposes. Furthermore, during the sway tests, the readings of some additional strain gauges were recorded by another datalogger, model *QuantumX* from *HBM*, sampling at a rate of 1 Hz, and the results were synchronised through a trigger input signal.

### Test programme and loading input

The main objective of the experimental campaign was to perform sway-tests on the specimens. Before and after the sway tests, all the specimens were subjected to snap-back tests, in which a load was applied at F1 location ( *cf*. Figure 34) using a fuse. After the fuse broke, the free vibration of the structures was measured through their displacements on the East side. These results were then used to estimate the vibration frequencies before and after the sway tests.

The following Table 5 reports the lists of the experiments, including both dynamic identification and quasi-static sway tests, performed on the four specimens.

**Table 5.** List and description of the tests performed.

Experiment Tag	Description	Maximum amplitude	Specimen	Purpose	Notes
b02	Dynamic Snap Back	F1 ~ 2 kN	uFrameM	Dynamic Identification	Pulling force at F1
b03	Dynamic Snap Back	F1 ~ 2 kN	uFrameM	Dynamic Identification	Pulling force at F1
c01	Monotonic Test (slab control)	120 mm	uFrameM	Quasi-static sway test	Displacement control using instruments on slabs
c02	Monotonic Test (frame control)	19 mm	uFrameM	Quasi-static sway test	Displacement control using instruments on frames
d01	Dynamic Snap Back	F1 ~ 2 kN	uFrameM	Dynamic Identification	Pulling force at F1
d02	Dynamic Snap Back	F1 ~ 2 kN	uFrameM	Dynamic Identification	Pulling force at F1
g01	Dynamic Snap Back	F1 ~ 2 kN	uFrameC	Dynamic Identification	Pulling force at F1. - D01: instrument HC3 not working properly; - D02: instrument HC3 not working properly; - D03: instrument HC3 replaced and working fine
l01	Cyclic Test (frame control)	120mm	uFrameC	Quasi-static sway test	Displacement control using instruments on frames
m02	Dynamic Snap Back		uFrameC	Dynamic Identification	Pulling force at F1
r02	Dynamic Snap Back	F1 ~ 2 kN	bFrameM	Dynamic Identification	Pulling force at F1
r11	Monotonic Test (frame control)	small cyclic tests (@1mm and 5mm) then push- over	bFrameM	Quasi-static sway test	Displacement control using instruments on frames. - D01 - cyclic and beginning of pushing - D02 - push-over test from initial "zero" position
r13	Dynamic Snap Back	F1 ~ 2 kN	bFrameM	Dynamic Identification	Pulling force at F1. - D01: 1st execution; - D02: 2nd execution. D01 and D02 are equivalent
s02	Dynamic Snap Back	F1 ~ 2 kN	bFrameC	Dynamic Identification	Pulling force at F1. - D01: sliding then fuse rupture at quite low force; - D02: sliding then fuse rupture, low force; - D03: fuse rupture, check data; - D04: fuse rupture, as wanted; - D05: fuse rupture, neglect the last part of the acquisition
t01	Cyclic Test (frame control)	135 mm	bFrameC	Quasi-static sway test	Displacement control using instruments on frames. - D01: pre-test at 1.694mm@Floor2; - D02: most of the test, then pause; - D03: acquisition with no movement; - D04: specimen brought back to initial position, then test at last amplitude.
u02	Dynamic Snap Back	F1 ~ 2 kN	bFrameC	Dynamic Identification	Pulling force at F1. -D01: empty; -D02: test data

The sway tests were conducted under displacement control. The monotonic tests were performed at a rate of 12 and 3 mm/min, with reference to the top storey lateral displacements, for the uFrame-M and BFrame-M, respectively. Following the results of preliminary numerical models, in the unbraced frames (uFrame-M and uFrame-C), the displacement applied in the 1 ^st^ storey was 54.5% of that applied in the top storey, simulating the displacement profile of the 1 ^st^ longitudinal translation vibration mode. Similarly, for the braced frames (BFrame-M and BFrame-C) a ratio of 59.0% was used.

**Figure 37.**
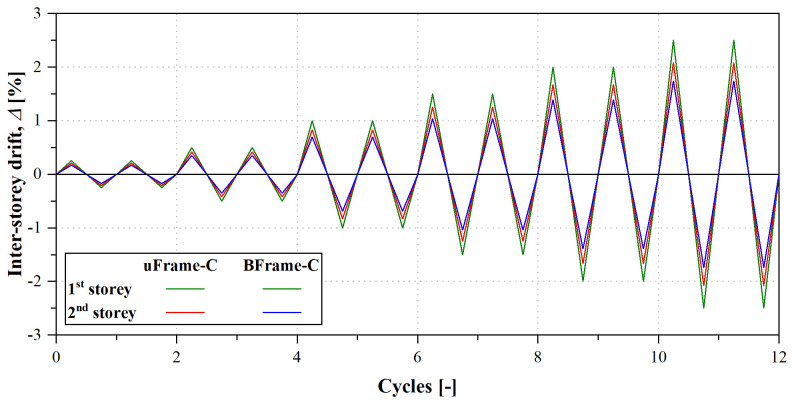
Displacement histories of the cyclic tests: inter-storey drift evolution.

The cyclic tests were conducted at a rate of ~18 mm/min, with respect to the top storey lateral displacement. Table 6 presents the displacement histories imposed to specimens uFrame-C and BFrame-C, and Figure 37 illustrates the evolution of the inter-storey drift with the cycles. The displacement protocol included 2 cycles for each applied displacement amplitude. The first 2 increments corresponded to a 0.25% drift of the bottom (governing) storey, while 0.50% increments were applied for the remaining cycles.

**Table 6.** Displacement histories used in cyclic tests.

Cycle	uFrame-C					BFrame-C				
	1 ^st^ Storey		2 ^nd^ Storey		Time (s)	1 ^st^ Storey		2 ^nd^ Storey		Time (s)
	Drift	Disp. (mm)	Drift	Disp. (mm)		Drift	Disp. (mm)	Drift	Disp. (mm)	
0	0.00%	0.00	0.00%	0.00	0	0.00%	0.00	0.00%	0.00	0
1	0.25%	8.00	0.21%	14.67	47	0.25%	8.00	0.17%	13.55	44
1	-0.25%	-8.00	-0.21%	-14.67	142	-0.25%	-8.00	-0.17%	-13.55	131
1	0.00%	0.00	0.00%	0.00	189	0.00%	0.00	0.00%	0.00	174
2	0.25%	8.00	0.21%	14.67	236	0.25%	8.00	0.17%	13.55	218
2	-0.25%	-8.00	-0.21%	-14.67	330	-0.25%	-8.00	-0.17%	-13.55	305
2	0.00%	0.00	0.00%	0.00	377	0.00%	0.00	0.00%	0.00	349
3	0.50%	16.00	0.42%	29.34	472	0.50%	16.00	0.35%	27.11	436
3	-0.50%	-16.00	-0.42%	-29.34	660	-0.50%	-16.00	-0.35%	-27.11	610
3	0.00%	0.00	0.00%	0.00	755	0.00%	0.00	0.00%	0.00	697
4	0.50%	16.00	0.42%	29.34	849	0.50%	16.00	0.35%	27.11	784
4	-0.50%	-16.00	-0.42%	-29.34	1038	-0.50%	-16.00	-0.35%	-27.11	959
4	0.00%	0.00	0.00%	0.00	1132	0.00%	0.00	0.00%	0.00	1046
5	1.00%	32.00	0.83%	58.68	1321	1.00%	32.00	0.69%	54.21	1220
5	-1.00%	-32.00	-0.83%	-58.68	1698	-1.00%	-32.00	-0.69%	-54.21	1569
5	0.00%	0.00	0.00%	0.00	1887	0.00%	0.00	0.00%	0.00	1743
6	1.00%	32.00	0.83%	58.68	2076	1.00%	32.00	0.69%	54.21	1918
6	-1.00%	-32.00	-0.83%	-58.68	2453	-1.00%	-32.00	-0.69%	-54.21	2266
6	0.00%	0.00	0.00%	0.00	2642	0.00%	0.00	0.00%	0.00	2441
7	1.50%	48.00	1.25%	88.03	2925	1.50%	48.00	1.04%	81.32	2702
7	-1.50%	-48.00	-1.25%	-88.03	3491	-1.50%	-48.00	-1.04%	-81.32	3225
7	0.00%	0.00	0.00%	0.00	3774	0.00%	0.00	0.00%	0.00	3486
8	1.50%	48.00	1.25%	88.03	4057	1.50%	48.00	1.04%	81.32	3748
8	-1.50%	-48.00	-1.25%	-88.03	4623	-1.50%	-48.00	-1.04%	-81.32	4271
8	0.00%	0.00	0.00%	0.00	4906	0.00%	0.00	0.00%	0.00	4532
9	2.00%	64.00	1.67%	117.37	5283	2.00%	64.00	1.39%	108.43	4881
9	-2.00%	-64.00	-1.67%	-117.37	6038	-2.00%	-64.00	-1.39%	-108.43	5578
9	0.00%	0.00	0.00%	0.00	6416	0.00%	0.00	0.00%	0.00	5927
10	2.00%	64.00	1.67%	117.37	6793	2.00%	64.00	1.39%	108.43	6276
10	-2.00%	-64.00	-1.67%	-117.37	7548	-2.00%	-64.00	-1.39%	-108.43	6973
10	0.00%	0.00	0.00%	0.00	7925	0.00%	0.00	0.00%	0.00	7322
11	2.50%	80.00	2.08%	146.71	8397	2.50%	80.00	1.74%	135.54	7757
11	-2.50%	-80.00	-2.08%	-146.71	9340	-2.50%	-80.00	-1.74%	-135.54	8629
11	0.00%	0.00	0.00%	0.00	9812	0.00%	0.00	0.00%	0.00	9065
12	2.50%	80.00	2.08%	146.71	10284	2.50%	80.00	1.74%	135.54	9501
12	-2.50%	-80.00	-2.08%	-146.71	11227	-2.50%	-80.00	-1.74%	-135.54	10372
12	0.00%	0.00	0.00%	0.00	11699	0.00%	0.00	0.00%	0.00	10808

In the first test, on specimen uFrame-M, the feedback displacements used by the control system were those measured at the slabs on the East side of the specimens (HS1 to HS4, *cf*. Figure 34 and Table 3). Following the shear connection failure on that specimen, due to the construction procedure described above ( *cf*. Section Construction Phases), this led to uneven displacements of the North and South GFRP frames. Beside changing the assembling procedure, in order to avoid similar possible issues, the displacements of the remaining specimens were controlled using the quantities measured by the instruments attached to the columns on the East side of specimens (HC1 to HC4, *cf*. Figure 34 and Table 3). In any case, it should be mentioned that, except for uFrame-M, the relative displacements between the slabs and the beams were negligible.

During the tests special care was taken to avoid the global collapse of the specimens which, due to their dimensions, could endanger the personnel and damage the test equipment. In that context, the BFrame tests were stopped after extensive local damage at the bracing connections, while the uFrame tests, for which there was no apparent signs of damage, were tested up to a 2% drift at the 1 ^st^ storey.

## Test observations

The present section includes a very brief description of the behaviour of the specimens, as observed in the experimental tests, to supplement this data paper. A comprehensive analysis and discussion of the results will be presented in future papers.

### uFrame-M

**Figure 38.**
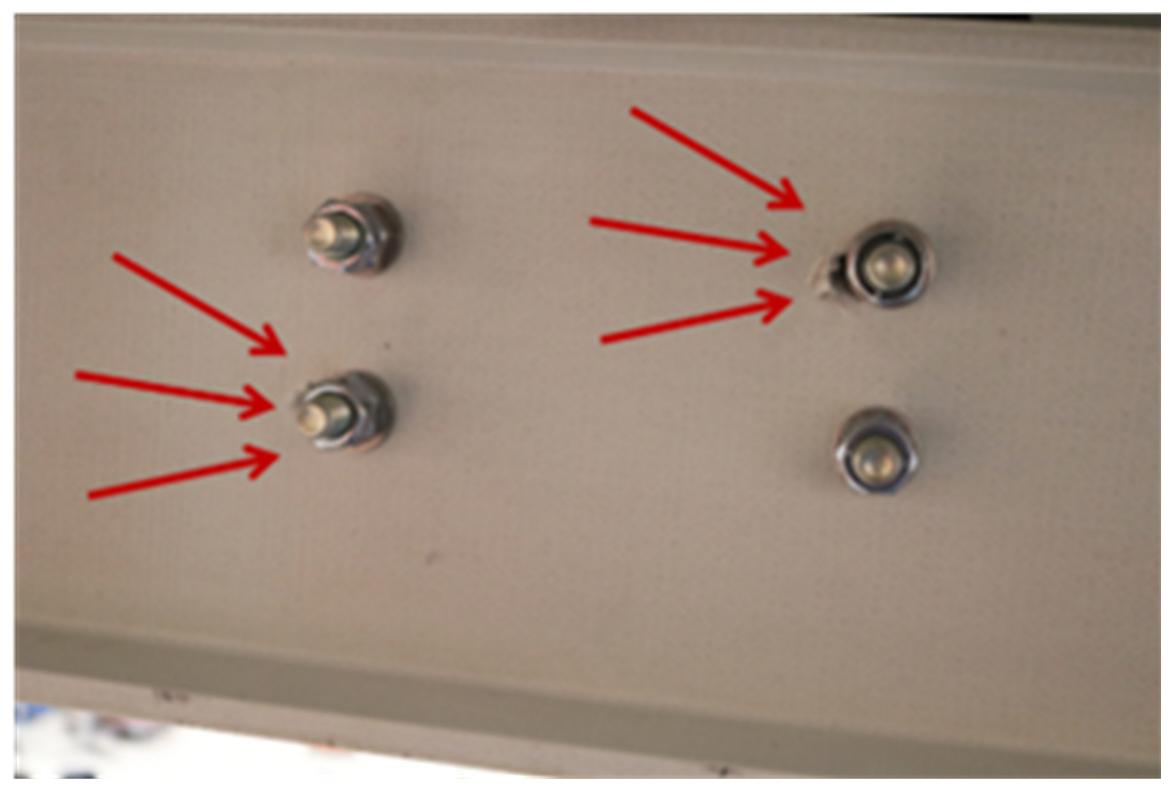
uFrame-M: damage at shear connection (post-test inspection).

**Figure 39.**
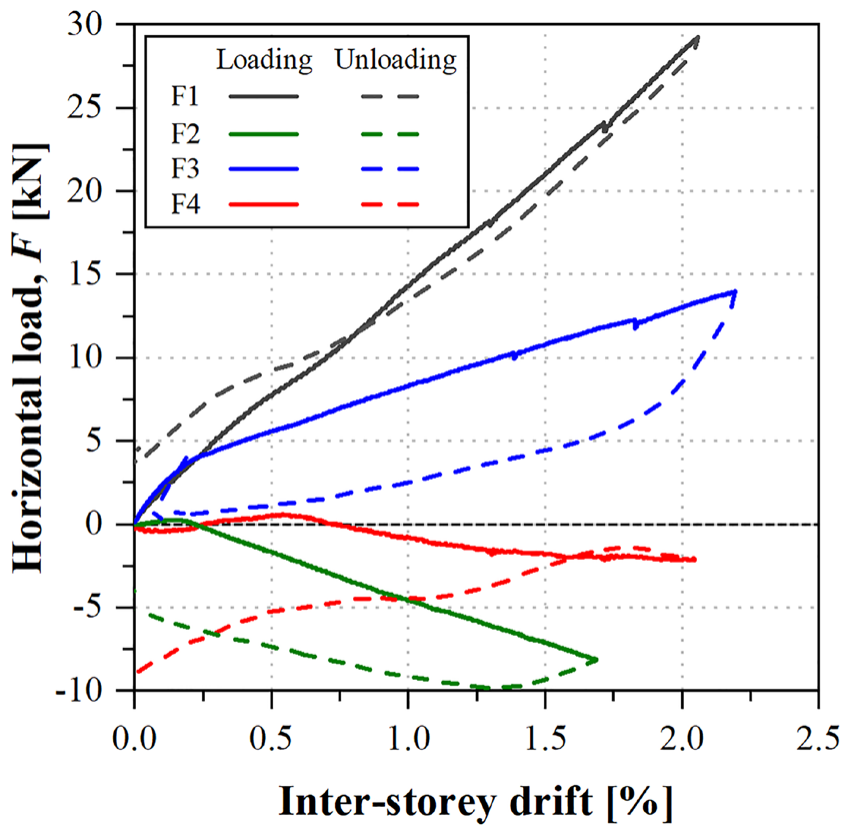
uFrame-M: load *vs*. inter-storey drift curves.

As mentioned, due to the different sequence of operations performed when assembling this first specimen, the shear connection between the concrete slab and the longitudinal beams of specimen uFrame-M was locally damaged before the test, which influenced its effectiveness. This was particularly relevant in the top North beam, as depicted in Figure 38. This damage was not noticed before the execution of the test, and resulted in an unexpected behaviour during the sway test of this specific specimen. Figure 39 shows the load *vs*. inter-storey drift curves obtained in that test. Each inter-storey drift was calculated based on the relative displacements measured at the composite columns on the East side of the specimens ( *cf*. Section Set-up and instrumentation) because, although in this particular specimen the control displacements were measured at the slab, there was sliding at the damaged shear connection.

The results show that, in order to apply the horizontal displacements, inverse loads needed to be applied: for the 1 ^st^ storey South lateral frame (F1) and 2 ^nd^ storey North frame (F3), the loads were applied in the direction of the displacements, as expected, while the other actuators (F2 and F4) applied the loads in the opposite direction. Moreover, the unloading paths diverged from the loading ones, except for actuator F1. The unexpected behaviour could only be attributed to the faulty shear connection, as no other signs of damage were detected in the structure, including during the post-test inspection.

In any case, it should be highlighted that the GFRP frame endured inter-storey drifts up to 2% with no apparent signs of damage. Furthermore, the results of the snap-back tests made before and after the sway tests yielded the same mode frequencies, showing that: (i) the structure was not damaged during the test, maintaining its original stiffness; and (ii) the damage at the shear connection occurred prior to the sway test (during construction) and/or it was affecting the behaviour only in case of high lateral loads.

### uFrame-C

Unlike what occurred for the uFrame-M specimen, the construction of the uFrame-C specimen followed the optimal construction protocol. In addition, and considering the issues found in the shear connection system between the longitudinal beams and the concrete floors in the previous experiment, additional steel plates were provided, strengthening the shear connection at the web of the beams. This ensured that there was no slip between the slabs and the frames; and, in fact, no signs of damage at the shear connection were registered during the test or in the post-test inspection.

Additionally, it is worth reminding that for this test (as well as for those on the braced frames), the control system was using the frame displacements measured at the East columns and not the slab displacements (initially preferred because those measurements were aligned with the loading).

**Figure 40.**
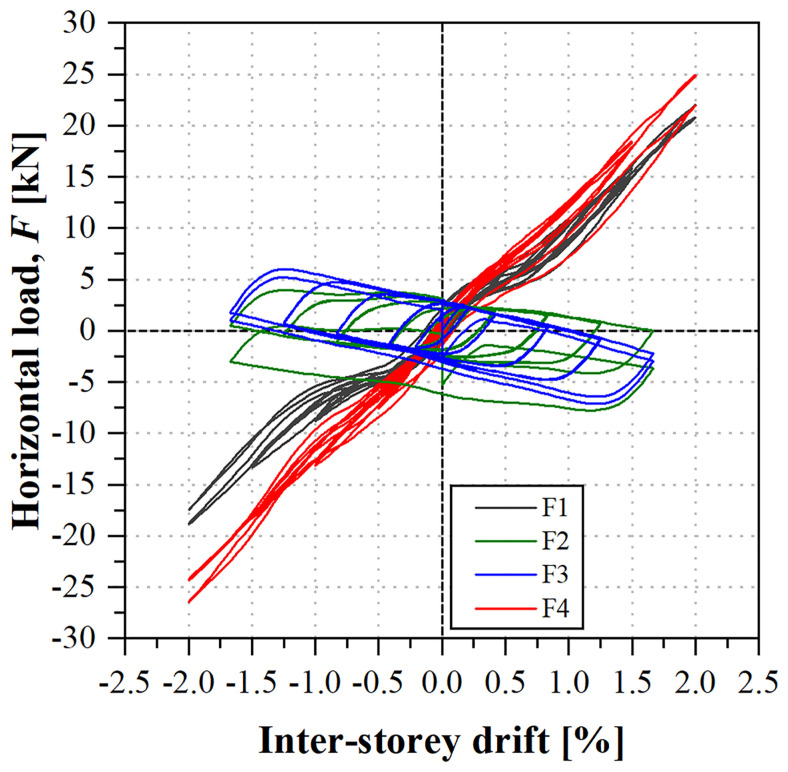
uFrame-C: load *vs*. inter-storey drift curves.

During the sway test, both lateral frames presented almost symmetric behaviour for positive and negative cycles, and for both lateral frames (North and South). The load *vs*. inter-storey drift curves, depicted in Figure 40, present a quasi-bi-linear behaviour: up to ~0.3% inter-storey drifts, load increased linearly with similar stiffness for the 1 ^st^ and 2 ^nd^ storeys. After that point, the 1 ^st^ storey loads (F1 and F4) continued to increase with the inter-storey drift but presented lower stiffness. On the other hand, the loads at the 2 ^nd^ storey started to decrease. This behaviour was observed throughout the test, for every cycle, resulting in relatively wide hysteretic curves for the 2 ^nd^ storey loads (F2 and F3), promoting energy dissipation.

During the tests, no signs of damage were observed, although cracking sounds were heard at the 1 ^st^ cycle of 1% inter-storey drift and at the 2 ^nd^ cycle of 1.5% drift (at the 1 ^st^ storey). Moreover, unlike what occurred in the previous specimen, there were slight period elongations, up to ~4%, when comparing the post-test and pre-test modal identifications, likely resulting from the ovalization of bolt holes in some connections. In any case, it should be stressed that these may only cause a minor loss of stiffness, and that the integrity of the structure was sound even after being subjected to cycling loading with -2%/+2% drifts.

### BFrame-M

**Figure 41.**
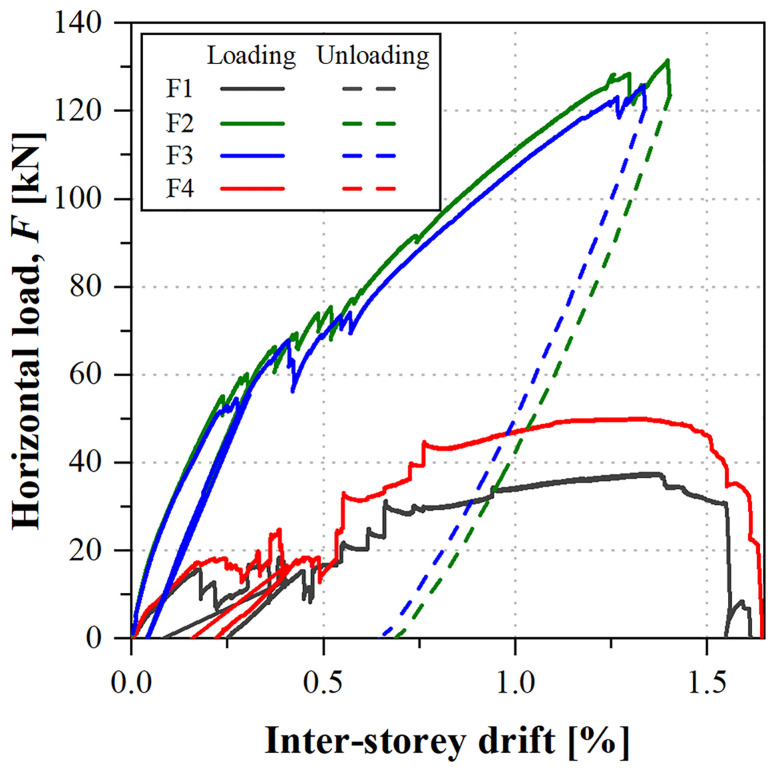
BFrame-M: load *vs*. inter-storey drift curves.

**Figure 42.**
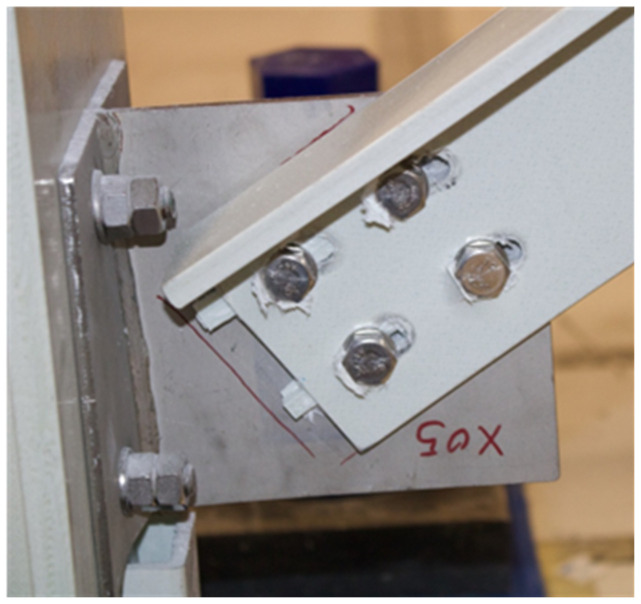
BFrame-M: failed bracing connection.

The braced frame presented, approximately, tri-linear behaviour, as shown in Figure 41. An initial stiffer stage, attributed to the friction of the bolted connections, was observed until ~0.04% inter-storey drifts were reached. After that stage, load increased quasi-linearly up to inter-storey drifts of ~0.18%. At this stage, crack sounds were heard, corresponding to the initiation of bearing failure at the bracing-to-column connections on the bottom floor. As the bearing failure progressed, loads continued to increase, with significantly lower stiffness and minute load drops until all 1 ^st^ storey bearing connections failed ( Figure 42) at ~0.75% inter-storey drifts. After that, a third stage ensued, with significantly reduced stiffness on the 1 ^st^ storey - F1 and F4 ( *i.e*., the actuators loading the first floor) presented an almost horizontal plateau, while the 2 ^nd^ storey stiffness remained approximately constant until the onset of bearing failure at its bearing connections (~1% inter-storey drift). Minute load drops were observed, as the bearing failure progressed and occurred on the different bracings, until all connections failed (ultimately, due to shear out), marking the end of the tests.

During the tests, the damages observed were limited to the bracing connections. The modal identification tests performed before and after the sway tests showed significant period elongation, as expected: before the sway tests, the bracings contribute to a significant increase of stiffness, while, after the sway test, the modal behaviour is similar to that of the unbraced structure, showing that the contribution of the damaged braces is negligible.

### BFrame-C

**Figure 43.**
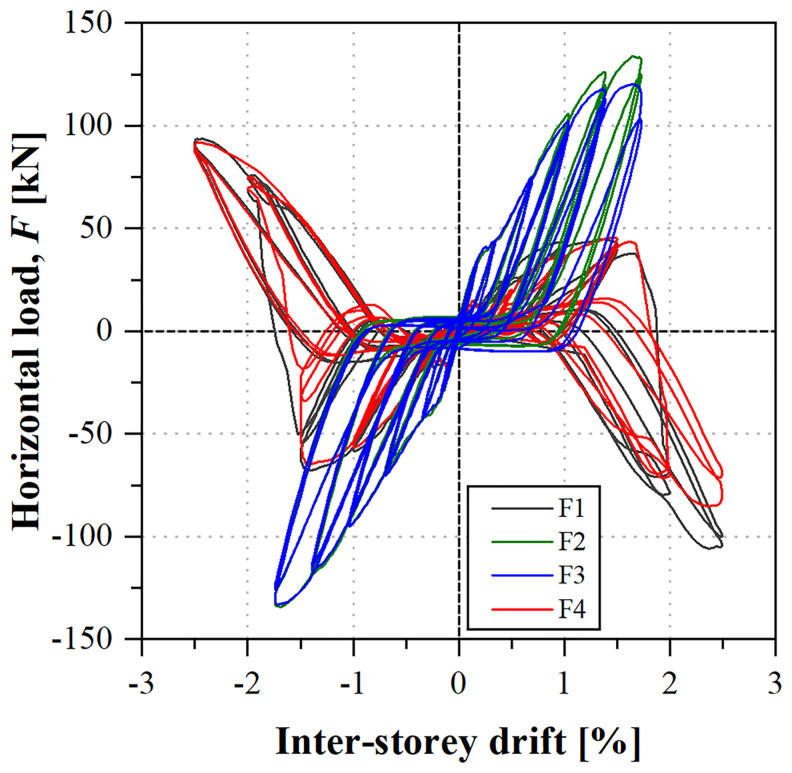
BFrame-C: load *vs*. inter-storey drift curves.

**Figure 44.**
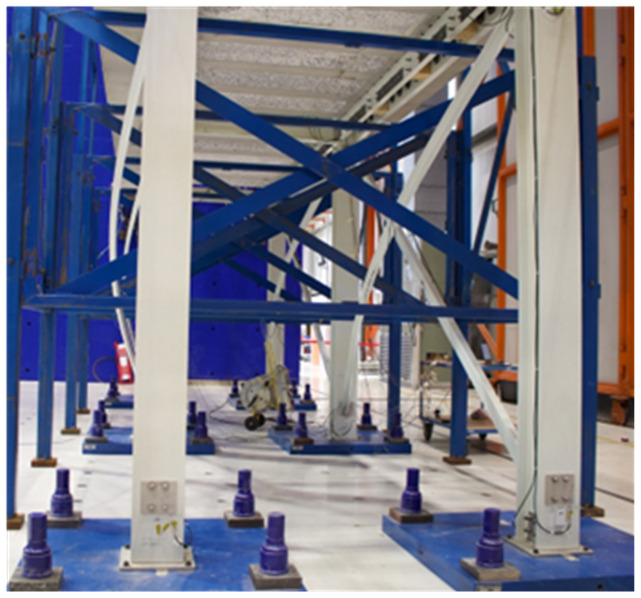
BFrame-C: significant buckling deformations of the compressed bracings.

The final specimen, a braced frame, was subjected to a cyclic test. Figure 43 shows the load *vs*. inter-storey drift curves, which are symmetric, both regarding the direction of the load and the lateral frames (South or North). The cyclic back-bone curves are similar to their monotonic counterparts ( *cf*. Figure 41), although the failure of the bracing connections occurred for slightly higher inter-storey drifts. It should be stressed that, due to buckling phenomena, depicted in Figure 44, the bracings contributed to the resistance of the frame only when loaded in tension. On the other hand, the results show that such buckling phenomena did not decrease their stiffness and strength, although significant permanent deformations were observed, mainly due to plastic deformations of the gusset plates. Nevertheless, upon load reversal, the significant out-of-plane displacements of the compressed bracings ( *cf*. Figure 44), stemming from flexural-torsional buckling phenomena, had to be inversed before those bracings could be subjected to tensile stresses, resulting in significant pinching ( *cf*. Figure 43).

As for the previous braced frame, visible signs of damage were limited to the bracing connections ( *cf*. Figure 42). Also, the modal identification tests yielded similar results, with significant period elongation after the sway tests, with the damaged structural response being analogous to that of the unbraced frames.

## Conclusions

This paper presented the data set of the sway tests performed on full-scale GFRP frame structures at the ELSA Reaction Wall of the Joint Research Centre physical research infrastructure of the European Commission. Comprehensive details were provided on the geometry and composition of the specimens, their construction procedure, measurement equipment, test setup and test procedures. This guarantees that the data set can be understood and used by third parties.

Future publications by the authors will focus on the detailed analysis and discussion of the results, including their relevance for the drafting of seismic design guidelines, compatible with the provisions of the future Eurocode for composite structures ^10^.

## Ethics and consent

Ethical approval and consent were not required.

## Data Availability

The paper briefly presents the Dissipate and Recentre (D&R) project, including all relevant information regarding the specimens, measuring equipment, test setup and test procedure. Future publications will include detailed analyses and discussion of the results. The dataset, gathering quantitative information and metadata, is made available by the authors in line with the requirement of the framework of the Open Access to JRC Research Infrastructures. The complete dataset can be downloaded at https://doi.org/10.5281/zenodo.15211782 ^21^ under a CC BY 4.0 license. In order to understand the structure of the shared dataset, the reader should refer to the Technical Report No JRC141264 ^19^. It is worth mentioning a short list of the main contents. These include (i) details of the specimens, (ii) applied loading patterns, (iii) details and calibration reports relative to the installed instrumentations (for further details on the calibration procedure refer to the Technical Report No JRC139347 ^22^). Besides, for each test, the dataset includes: (i) raw data as recorded by the acquisition system of the ELSA lab, (ii) processed data for an easier access to the output of the monitoring, (iii) graphical representation of the main response quantities, (iv) photo sequences taken during the execution of the testing protocol.
