# Supplementary material for: Dissipate and Recentre (D&R) experimental campaign on real scale GFRP composite frame structures: data set
Source: Open Res Eur. 2026 Jan 14;5:308. Originally published 2025 Oct 8. [Version 2] doi: 10.12688/openreseurope.20500.2 (PMC12891970; doi:10.12688/openreseurope.20500.2)
Supplement: Peer review history

# Reviewer report: Reviewer response for version 2

**DOI:** 10.21956/openreseurope.24516.r68655

**Published:** 2026-02-11

**Authors:** Angelo Savio Calabrese

**Affiliations:** 1 Politecnico di Milano, Milan, Italy

The repository provides a clearly curated experimental dataset that is presented with a strong emphasis on transparency, traceability, and practical reusability. The documentation of the testing campaign, including data organization, metadata, and supporting files, allows the reader to readily understand how the data were generated and how they can be accessed and reused without ambiguity. The authors demonstrate good practice in openly reporting experimental choices, test conditions, and deviations encountered during the campaign, which enhances the credibility of the dataset and supports reproducibility. Overall, the work appears robust and well prepared for reuse by both academic and applied researchers, and it represents a useful contribution within the scope and objectives of a data article.

 Given the completeness and level of detail of the report, the comments below are solely intended to invite the authors to enrich the article by sharing practical recommendations derived from the experience gained during the experimental campaign. As is often the case when an experimental program is completed, hindsight may highlight aspects that could have been addressed differently or improved if the study were to be repeated. In this spirit, the following comments are intended to encourage the authors to reflect on and document this “lessons learned” perspective, based on their direct experimental experience.

 1 - The authors are kindly asked to clarify the rationale adopted for the structural design of the tested frame, particularly in relation (if any) to the recently issued PD CEN/TS 19101:2022, *“Design of fibre-polymer composite structures".* It would be useful to understand whether the indications provided by this document were considered at any stage of the design process, and how applicable the authors judge such design guidance to be when confronted with the practical execution of a real, multi-storey GFRP structure. Given that this frame represents one of the few early examples of a multi-storey structure realized using structural GFRP profiles, the authors’ critical assessment of the applicability and limitations of current design recommendations would be of clear value to future designers and researchers.

 2 - The discussion of the experimental results would benefit from a final section in which the authors summarize their experience-based suggestions on how the design of similar GFRP structures could be improved and made more effective. In this respect, the structural connections appear to represent a "weak link" of the overall system performance. The authors are encouraged to discuss how connection design could be enhanced, and whether alternative solutions, such as fully bonded or hybrid bonded–bolted connections, may provide improved performance compared to the configurations tested in the present study.

 3 - It would be useful to assess whether a direct correlation can be established between the pin-bearing strength of the GFRP material reported in Table 1 and the observed failure of the structural connections, particularly when connection failure governs the global crisis of the structure. Clarifying this relationship, even at a qualitative or semi-quantitative level, would improve the interpretability of the dataset and support its reuse for connection modelling and design-oriented studies.

 4 - Both local and global buckling of GFRP structural elements appear to limit the exploitation of the full capacity of the tested frame. The authors are encouraged to comment on potential strategies, based on their experimental experience, to mitigate buckling phenomena in similar structures (at the design stage). Such insights would further enhance the practical value of the dataset for researchers and practitioners interested in the development of efficient and reliable GFRP framing systems.

Are sufficient details of methods and materials provided to allow replication by others?

Yes

Is the rationale for creating the dataset(s) clearly described?

Yes

Are the datasets clearly presented in a useable and accessible format?

Yes

Are the protocols appropriate and is the work technically sound?

Yes

Reviewer Expertise:

My research has focused in particular on the static and fatigue behavior of composite materials for structural applications, with special attention to both fully composite structures and externally bonded strengthening systems applied to existing steel, concrete, and masonry structures.

I confirm that I have read this submission and believe that I have an appropriate level of expertise to confirm that it is of an acceptable scientific standard.

# Reviewer report: Reviewer response for version 2

**DOI:** 10.21956/openreseurope.24516.r68657

**Published:** 2026-02-10

**Authors:** Mohammadreza Vafaei

**Affiliations:** 1 Universiti Teknologi Malaysia, Johor, Malaysia

The manuscript presents experimental results from four large-scale composite frame structures subjected to gravity and cyclic loading. The paper is well-written and offers valuable insights into the fabrication, testing protocols, and subsequent performance of these systems. However, additional details regarding the design methodology and specific failure modes are required to improve the clarity and reproducibility of the study. Addressing the following comments will enhance the quality of the manuscript.

**Specific Comments**

**1. Design Methodology and Loading: **The rationale behind the member sizing (beams, columns, and braces) requires further elaboration. Please include the specific design concepts and standards used. Specifically, provide the values for the design dead loads, live loads, and seismic parameters that governed the selection of these member sizes.

**2. Connection Details: **Please provide a more granular description of the connection design process. It would be beneficial to include design method and standard used to determine the number of bolts and bolt dimensions for both beam-to-column and brace-to-column connections.

**3. Beam-to-Column Connection Behavior: **The detailing employed for the beam-to-column connections suggests a semi-rigid response. Please justify the selection of this specific detail. Was this intended to model a specific existing construction practice?

**4. Bearing Failure Analysis: **Figures 38 and 42 appear to indicate bearing failure. Please clarify if this specific failure mode was anticipated and accounted for during the design stage (e.g., via checking bearing limit states vs. bolt shear).

**5. Work-Point Eccentricity: **Figure 10 shows that the brace centerline does not intersect with the column centerline (work-point eccentricity). This configuration imposes additional bending moments on the columns. Please explain the reasoning behind this detailing choice and discuss how the additional moment demands were accounted for in the column design.

**6. Suitability of Single Angle Braces: **Single angle braces are generally not recommended for Special Concentrically Braced Frames (SCBF) due to their susceptibility to flexural buckling and potential for poor cyclic performance. Please justify the use of single angles in this study. Furthermore, please provide the slenderness ratio.

**7. Strain Data: **The current manuscript lacks measured strain data. The inclusion of strain histories is beneficial to better understand the cyclic response of the frames.

Are sufficient details of methods and materials provided to allow replication by others?

Partly

Is the rationale for creating the dataset(s) clearly described?

Yes

Are the datasets clearly presented in a useable and accessible format?

Yes

Are the protocols appropriate and is the work technically sound?

Yes

Reviewer Expertise:

Seismic design and retrofitting, Vibration control , Structural health monitoring

I confirm that I have read this submission and believe that I have an appropriate level of expertise to confirm that it is of an acceptable scientific standard.

# Reviewer report: Reviewer response for version 2

**DOI:** 10.21956/openreseurope.24516.r68076

**Published:** 2026-01-19

**Authors:** Marco Civera

**Affiliations:** 1 Polytechnic of Turin, Turin, Piedmont, Italy

The revised version is fully compliant.

Are sufficient details of methods and materials provided to allow replication by others?

Yes

Is the rationale for creating the dataset(s) clearly described?

Yes

Are the datasets clearly presented in a useable and accessible format?

Yes

Are the protocols appropriate and is the work technically sound?

Yes

Reviewer Expertise:

Structural Health Monitoring and Damage Assessment

I confirm that I have read this submission and believe that I have an appropriate level of expertise to confirm that it is of an acceptable scientific standard.

# Reviewer report: Reviewer response for version 1

**DOI:** 10.21956/openreseurope.22184.r62457

**Published:** 2025-11-13

**Authors:** Haitham Al-Thairy

**Affiliations:** 1 University of Al-Qadisiyah, Al Diwaniyah, Al Diwaniyah, Iraq

This article presents a data set of a full-scale experimental test of four GFRP two bay two story frames under static and quasi cyclic static load. Two of the frames are unbraced while the other two are braced against lateral movement. The frames are subjected to a displacement control loading up to global or local damage of the frames on a west side of the farms and the inert-story drift were measured at each story level at the east side of the frames. The experimental programed was described in terms of the material, geometry, test setup, loading scheme data collection and recording devices Also, a primarily data were presented in terms of the load- inert-story drift for each frame considered in the tests. The research project is very interesting in terms of qualifying the lateral resilience of GFRP frames under seismic events and the determine the potential of using advantageous properties of GFRP such as the high tensile strength, low density and low inertia force in reducing the damage of structures and saving the human lives. However, the following comments are suggested by the reviewer:

1.  A pre cast concrete slab is attached to the GFRP beams to simulate the equivalent vertical loads of a real application, and allowing to anchor the actuators as suggested by the authors. On the other hand, it has been mentioned in another place of the manuscript that " It should be stressed that the concrete slabs are not part of the conceptual design of the specimens (in an actual all-composite structure, the floors would likely be made of composite sandwich panels), serving only as a mean to apply vertical (dead) and horizontal (connected to the actuator) loads without suffering any damage during the tests". However, the existence of the concrete slabs significantly affects the behavior of the frames in terms of increasing the total weight of the frame and requiring additional details such as shear connectors, bolts, etc. These effects increase the potential failure of the frames globally due to inertia effects and locally due to failure of the connection's details between the slab and the beams
2. A displacement history imposed to specimens to simulate the tests at a rate of18 mm/min. However, it is more rational to apply displacement time history which is normally much higher the rate to simulate a real earthquake displacement or acceleration.
3. Please explain why hysteretic curves the top stories ( F2, F3)  forces are in a reverse direction of the bottom stories ( F1, F4) ?
4. Please present the symbols of the forces in a sequential order (i.e. F1, F2, ..) in the legend of the curves   

Are sufficient details of methods and materials provided to allow replication by others?

Yes

Is the rationale for creating the dataset(s) clearly described?

Partly

Are the datasets clearly presented in a useable and accessible format?

Yes

Are the protocols appropriate and is the work technically sound?

Partly

Reviewer Expertise:

behavior and failure of GFRP RC members , dynamic analysis of structures, developing analytics methods of the static and dynamic analysis of structures particularly structures under impact and blast.

I confirm that I have read this submission and believe that I have an appropriate level of expertise to confirm that it is of an acceptable scientific standard, however I have significant reservations, as outlined above.

# Response

**Published:** 2026-01-02

**Authors:** Jose Gonilha

The authors thank the reviewer for the overall positive assessment of the paper. Below, you can find a detailed reply to each of your specific comments:

**Reply 1:** The authors agree that the presence of the concrete slab influences the behaviour of the frame, particularly due to the increase in total weight. However, we would like to raise the attention of the reviewer to the fact that this was precisely the objective of using the concrete slabs: to simulate the weight of a floor system used in composite structures (e.g., sandwich panels), the weight of other permanent loads (partition walls, furniture, etc), and the quasi-permanent part of the live loads, thereby encompassing the weight used in the seismic design combination. Additionally, the authors acknowledge that local failure of the connection details is a possibility, which was prevented by overdesigning these connections – note that the localized failure in the shear connection that was observed (in a single specimen) was due to an error in the construction process, and did not occur during the test. Moreover, as stated in the manuscript, the “design of the slabs guaranteed that there was no concrete-GFRP composite action in the vicinity of the supports, and that the slabs did not transmit bending moments from one span to the other”; this minimizes the composite action between the beams and the slabs, localizing it at the centre of the spans, which play a smaller role in the sway behaviour of the frames.

**Reply 2:** The use of quasi-static experimental tests to simulate the response of structures to earthquake actions is a well-established practice (e.g., https://doi.org/10.1193/1.1585869). This technique allows the cost-effective assessment of structural systems, particularly at large scales, contributing with a wealth of data that allows to determine their seismic behaviour. It should also be noted that the data obtained with sway-tests closely relates with that of seismic design procedures, namely P-delta analysis. Furthermore, the outcomes of cyclic tests are recognised as the most suitable for the estimation of structural capacity, and the calibration of numerical models, also due to the valuable information about the stability of structural response and energy-dissipation capacity.

**Reply 3:** The different signs of the top and bottom storey loads are attributed to the fact that the displacement history imposed on the specimens diverged, at some point, from the first modal configuration of the specimens. As mentioned in the paper, the modal shapes were obtained from preliminary numerical models. In practice, when the stiffness of the connections change, in this case due to surpassing the friction limit, the modal characteristics of the structure change. This can result in the imposition of an unnatural deformations, namely regarding the top/bottom displacement ratios. This discussion has not been included in this paper because this is a data paper, focused on providing all experimental data and information about the test results. Future papers will focus on the analysis and discussion of the results. It can be added that the authors know this effect could have been avoided adopting different control strategies. For instance, imposing displacement-controlled cycles at the second floor, while applying force-controlled cycles (e.g. imposing a constant ratio between the forces at the first and at the second stories equal to 0.5) would have avoided the story-forces to counteract each other. On the other hand, such a loading strategy would have activated a soft-story mechanisms preventing the possibility to achieve the maximum capacity of the second storey.

**Reply 4:** The plots (Figures 39, 41, 40, and 43) have been changed following the reviewer’s recommendation.

# Reviewer report: Reviewer response for version 1

**DOI:** 10.21956/openreseurope.22184.r62458

**Published:** 2025-11-03

**Authors:** Marco Civera

**Affiliations:** 1 Polytechnic of Turin, Turin, Piedmont, Italy

The repository described by this report represents a valuable dataset of full-scale GFRP frames, with rich instrumentation and clear test logs. The note is strong, and the experimental data are reusable and of interest to the scientific and technical community.

 The dataset, specimens, set-up, instrumentation, and test programs are all succinctly, transparently, and efficiently described.

 Most importantly, the use of full-scale models is highly relevant for accounting for potential scale effects that are generally dismissed in scaled-down laboratory replicas.

 The investigated frames are realistic, also in their braced/unbraced variants and (nominal) mass distribution.

 Notes on apparent damage (or lack thereof) are coherent and explicitly detailed. The bracing connection failure taxonomy is also clear and understandable.

 The hardware details are good and explicit.

 Issues such as connection assembly problems are openly and honestly reported (see uFrame-M). Countermeasures adopted for other specimens are clearly detailed.

 The instrumentation is particularly rich, including calibration reports.

 The level of detail in the test log is also laudable.

 The dataset directly enables several reuse scenarios, from Finite Element model calibration for GFRP frames (also accounting for material orthotropy and bolted joints), pinched hysteresis models for braced systems, connection fragility/limit states, and validation of control strategies.

 All being said, the dataset is well-structured and a very good contribution. Especially since GFRP offers an attractive package for building frames: very high specific strength at a fraction of steel’s weight (thus requiring smaller foundations and producing a lower seismic mass), competitive specific stiffness, low thermal conductivity for reduced thermal bridging, and outstanding corrosion resistance that virtually eliminates chloride- or moisture-driven deterioration.

 Most importantly, Pultruded members deliver consistent, factory-controlled quality and enable rapid, lightweight construction. Well-detailed GFRP systems can provide a stable cyclic response with minimal residual deformation. Hence, in the near future, GFRP unlocks durable, low-maintenance, fast-to-install structures with compelling whole-life benefits; this report, the dataset it describes, and the funded research go in the right direction for these aims.

Are sufficient details of methods and materials provided to allow replication by others?

Yes

Is the rationale for creating the dataset(s) clearly described?

Yes

Are the datasets clearly presented in a useable and accessible format?

Yes

Are the protocols appropriate and is the work technically sound?

Yes

Reviewer Expertise:

Structural Health Monitoring and Damage Assessment

I confirm that I have read this submission and believe that I have an appropriate level of expertise to confirm that it is of an acceptable scientific standard.

# Response

**Published:** 2026-01-02

**Authors:** Jose Gonilha

The authors greatly appreciate the comprehensive assessment of the paper made by the reviewer.
